# RG100204, A Novel Aquaporin-9 Inhibitor, Reduces Septic Cardiomyopathy and Multiple Organ Failure in Murine Sepsis

**DOI:** 10.3389/fimmu.2022.900906

**Published:** 2022-06-14

**Authors:** Shireen Mohammad, Caroline E. O’Riordan, Chiara Verra, Eleonora Aimaretti, Gustavo Ferreira Alves, Klaus Dreisch, Johan Evenäs, Patrizia Gena, Angela Tesse, Michael Rützler, Massimo Collino, Giuseppe Calamita, Christoph Thiemermann

**Affiliations:** ^1^ William Harvey Research Institute, Queen Mary University of London, London, United Kingdom; ^2^ Department of Clinical and Biological Sciences, University of Turin, Turin, Italy; ^3^ Department of Neurosciences “Rita Levi Montalcini”, University of Turin, Turin, Italy; ^4^ Red Glead Discovery Akiebolag (AB), Lund, Sweden; ^5^ Department of Biosciences, Biotechnologies and Biopharmaceutics, University of Bari “Aldo Moro”, Bari, Italy; ^6^ Nantes Université, Instite National de la Santé et de la Recherche Médicale (INSERM), Centre National de la Rescherche Scientifique (CNRS), l’institut du Thorax, Nantes, France; ^7^ Department of Biochemistry and Structural Biology, Lund University, Lund, Sweden; ^8^ Apoglyx Akiebolag (AB), Lund, Sweden

**Keywords:** aquaporin (AQP), sepsis, cecal ligation and puncture, inflammation, multiple organ failure

## Abstract

Sepsis is caused by systemic infection and is a major health concern as it is the primary cause of death from infection. It is the leading cause of mortality worldwide and there are no specific effective treatments for sepsis. Gene deletion of the neutral solute channel Aquaporin 9 (AQP9) normalizes oxidative stress and improves survival in a bacterial endotoxin induced mouse model of sepsis. In this study we described the initial characterization and effects of a novel small molecule AQP9 inhibitor, RG100204, in a cecal ligation and puncture (CLP) induced model of polymicrobial infection. *In vitro*, RG100204 blocked mouse AQP9 H_2_O_2_ permeability in an ectopic CHO cell expression system and abolished the LPS induced increase in superoxide anion and nitric oxide in FaO hepatoma cells. Pre-treatment of CLP-mice with RG100204 (25 mg/kg p.o. before CLP and then again at 8 h after CLP) attenuated the hypothermia, cardiac dysfunction (systolic and diastolic), renal dysfunction and hepatocellular injury caused by CLP-induced sepsis. Post-treatment of CLP-mice with RG100204 also attenuated the cardiac dysfunction (systolic and diastolic), the renal dysfunction caused by CLP-induced sepsis, but did not significantly reduce the liver injury or hypothermia. The most striking finding was that oral administration of RG100204 as late as 3 h after the onset of polymicrobial sepsis attenuated the cardiac and renal dysfunction caused by severe sepsis. Immunoblot quantification demonstrated that RG100204 reduced activation of the NLRP3 inflammasome pathway. Moreover, myeloperoxidase activity in RG100204 treated lung tissue was reduced. Together these results indicate that AQP9 may be a novel drug target in polymicrobial sepsis.

## Introduction

Sepsis and septic shock result in multiple-organ failure that can lead to death (1). Aquaporins (AQPs) are a family of membrane channel proteins which are responsible for facilitating diffusion of water as well as glycerol across the plasma membrane (2). Briefly, there are 13 AQPs in mammals which are important for the regulation of water movement in and out of the cell. Some AQPs can also facilitate passive movement of glycerol and other small solutes including urea and hydrogen peroxide (H_2_O_2_) cross the cell membrane. Aquaporins can regulate crucial key mechanism of sepsis and hence could be promising drug targets (3).

In physiology, AQP9 is the principal facilitator of glycerol uptake into hepatocytes, for entry into gluconeogenesis, especially during fasting (4, 5). Previously described AQP9 functions in leukocytes include maturation of dendritic cells (6), glycerol metabolism in CD8^+^ T memory cells, and an involvement in locomotion in neutrophils (7, 8). Moreover, when compared to healthy controls, neutrophils from patients with SIRS show increased AQP9 expression (9).

In addition to facilitating glycerol transmembrane diffusion, AQP9 is also permeable to water, and H_2_O_2_ (10–12). There is now increasing evidence that H_2_O_2_ permeability mediated by different AQP isoforms contributes to redox signaling in diverse cellular processes including locomotion (13, 14), growth factor signals (15), cytokine receptor signals (16), and toll-like receptor signal (17). Since AQP9 plays a role in metabolism and inflammation, it may also play a role in the progression of sepsis. Indeed, a recent study points towards a role for AQP9 in the pathophysiology of sepsis: when compared to wild-type mice, *Aqp9* ^-/-^ knockout mice challenged with a lethal dose of LPS had a longer survival time and 25% of these mice recovered fully. *Aqp9* ^-/-^ knockout mice subjected to endotoxemia also showed reduced iNOS expression (and NO formation) and COX-2 expression, secondary to a reduced expression/activation of NF-ĸB RelA/p65 in the kidney, aorta, liver, and heart compared to wild-type mice induced with LPS (18). The inflammasome component NLRP3 is upregulated in sepsis which leads to the cleavage of pro-caspase-1 to caspase-1, and subsequently maturation and release of the proinflammatory cytokine, IL-1β. As an intriguing hypothesis, a requirement of water influx *via* AQPs in the activation of the inflammasome has been introduced (19). However, experiments supporting this idea involved harsh osmotic conditions, and sometimes the use of the pleiotropic and cytotoxic AQP blocker HgCl_2_ (19–21). Therefore, this idea requires further investigation. The development of specific aquaporin-inhibitors that are suitable for *in vivo* experiments has however been historically difficult (22, 23). We now describe the first use of a new AQP9 inhibitor, RG100204, in a cecal ligation and puncture (CLP) mouse model of peritoneal infection and systemic inflammation.

## Methods

### RG100204

The compound RG100204 is described as example 29 in the patent US20190127360 (24) The patent contains description of the used synthetic route and the LC-MS and ^1^H NMR data.

### RG100204 Formulation for Oral Administration (25 mg/kg BW)

PEG 400 (polyethylene glycol 400, Acros Organics), Labrasol^®^ (caprylocaproyl polyoxyl-8-glycerides, Gattefossé), and Kolliphor^®^ EL (polyoxyl-35 castor oil, Sigma Aldrich) were mixed to a ratio of 50:30:20 w%. The components were mixed by stirring on a rolling mixer. Dry powder of RG100204 was solubilized in this blank vehicle to a concentration of 10 mg RG100204 per g vehicle. The formulation was homogenized by ultrasonication.

### Cell Based Assays

Flp-In™-CHO Cells were purchased from Invitrogen (ThermoFisher) and modified as described previously (5, 11). Water permeability was measured as cell shrinking in response to extracellular sucrose addition, as described previously (5). Glycerol permeability was measured in the same way, except for the addition of 500 mM glycerol to the assay incubation buffer. The incubation time in glycerol buffer varied between 5 and 50 minutes, depending on the position of each well within a 96-well plate. No effect of incubation time was noted. Glycerol efflux was induced by replacing 250 mM of the extracellular glycerol with 250 mM sucrose, by 1:1 dilution with 500 mM sucrose containing buffer. Measurements of H_2_O_2_ permeability were conducted as described previously (11).

% Permeability Was Calculated as:


$$\%permeability=1-\frac{K\;\left[AQP9\;control]-K\;[AQP9\;inhibitor\right]}{K\;\left[AQP9\;control\right]\;-K\;\left[CHO\right]}$$

Whereby *K* corresponds to the rate constant, obtained from fitting the fluorescence intensity recordings after sucrose, and H_2_O_2_ addition, respectively, to a one-phase exponential function. [*AQP9 control*] describes AQP9 expressing cells incubated in 1% DMSO, the inhibitor vehicle; [*AQP9 inhibitor*] describes cells incubated at respective inhibitor concentration; and [*CHO*] describes recordings from CHO control cells, or CHO HyPer-3 (25) expressing control cells, respectively, that do not express ectopic AQP9.

### NO and Superoxide Anion Measurements

Rat hepatoma FaO cells [The European Collection of Authenticated Cell Cultures (ECACC)] were grown in Coon’s modified Ham’s F12 with 10% fetal bovine serum (FBS) until 80% confluence. Cell incubations were made in humidified atmospheric air at 37°C with CO_2_ added to 5%. Cell monolayers composed of about 5x10^5^ cells were exposed for 6 h at 37°C to the culture medium (1% DMSO) containing 25 µM RG100204, 1 µg/mL LPS (from Escherichia coli 0111:B4; Sigma) or 25 µM RG100204 plus 1 µg/mL LPS. For the basal condition cells were exposed to the culture medium containing the vehicle alone (1% DMSO). At the end of the treatment cell samples of each condition were harvested and handled to prepare the spin traps for the subsequent nitric oxide (NO) and superoxide anion $\left({\text{O}}_{2}^{-}\right)$ measurements as described below.

The cytotoxicity of RG100204 in FaO cells was evaluated by MTT (3-(4,5-dimethylthiazol-2-yl)-2,5-diphenyltetrazolium bromide) cell viability assay (Biotium, Milan, Italy). FaO cells were seeded in a 96-well plate and incubated 24 h in humidified atmospheric air at 37°C with CO_2_ added to 5%. After treated with the vehicle alone (1% DMSO) or 25 μM RG100204 (in 1% DMSO) for 24 h, cells were analyzed by adding MTT. The colorimetric absorbance was measured by an iMark™ microplate reader (BIO-RAD, Segrate, Italy) at 490 nm.

Electron paramagnetic resonance (EPR) for NO and ${\text{O}}_{2}^{-}$ measurements with the spin traps of FaO cells prepared as above was carried out as previously described (18). Briefly, Fe^2+^ diethyldithiocarbamate (Fe(DETC)_2_) was used as spin trap to evaluate NO production. For this purpose, Na-(DETC) (3.6 mg; Sigma) and FeSO_4_-7H_2_O (2.3 mg; Sigma) were dissolved separately in equal volumes of ice-cold Krebs–Hepes buffer or distilled water, respectively, each one bubbled with nitrogen gas. The solutions were mixed to obtain a pale yellow-brown opalescent colloid Fe(DETC)_2_ solution (0.4 mM), which was used immediately to incubate the FaO cells (45 min at 37°C). After incubation, the spin trap solution was removed, and cells were scraped off and frozen in liquid nitrogen.

To evaluate extracellular and intracellular production of ${\text{O}}_{2}^{-}$ , FaO cells were incubated in a Krebs-Hepes solution containing 500 µM of 1-hydroxy-3methoxycarbonyl-2,2,5,5-tetramethylpyrrolidin (CMH; Noxygen, Denzlingen, Germany), 25 µM deferoxamin (Sigma) and 5 µM DETC (Sigma) for 45 min at 37°C. To measure the extracellular superoxide anion concentration, CMH solution was removed after the incubation step, and frozen in liquid nitrogen. Cells were then scraped off from the culture plate and frozen in liquid nitrogen. All the samples were analyzed using a table-top x-band spectrometer Miniscope (Magnettech, MS5000, Berlin, Germany). Recordings were made in liquid nitrogen, using a Dewar flask.

The instrument settings were 10 mW of microwave power, 1 mT of amplitude modulation, 100 kHz of modulation frequency, 180 s of sweep time and 3 scans for NO measurements, sweep time 60 s, and 3 scans for superoxide anion measurements. Signals were quantified by measuring the total amplitude of the spectra obtained, after correction of baseline for NO evaluation or by calculating the height of the central peak of the spectra for superoxide anion measurements.

### Ethical Statement

All animal protocols in this study were approved by the Animal Use and Care Committee of Queen Mary University of London (QMUL), in accordance with the Home Office Guidance on the Operation of Animals (Scientific Procedure Act 1986), published by Her Majesty’s Stationary Office and the Guide for the Care and Use of Laboratory Animals of the National Research Council and were approved by the Animal Welfare Ethics Review Board of QMUL. All research was conducted under the U.K. home office project license number: PC5F29685.

### Animals

This study was carried out on 10-week-old, male C57BL/6 mice (Charles River Laboratories UK Ltd., Kent, UK) weighing 20-30 g kept under standard laboratory conditions. The animals were allowed to acclimatize to laboratory conditions for at least one week before undergoing experiments. Six mice were housed together in ventilated cages lined with absorbent bedding material. Tubes and chewing blocks were placed in all cages for environmental enrichment. All animals were subjected to 12-h light and dark cycles and the temperature was maintained at 19-23°C. All animals had access to a chow diet and water *ad libitum*. The cages were cleaned approximately every three days, with water being changed daily. Research staff inspected the animals each day for any signs of illness or abnormal behavior.

### Cecal Ligation and Puncture (CLP) Model

CLP surgery was performed on 10-week-old C57BL/6 mice. Sham-operated mice underwent sham surgery, which involved the same treatment; however, these mice were not subjected to ligation and puncture of the caecum. Before starting surgery, mice were injected with buprenorphine (0.05 mg/kg i.p.) to provide analgesia. Mice were initially anaesthetized with 3% isoflurane and 1% oxygen and maintained under anaesthesia throughout surgery with 2% isoflurane and 1% oxygen *via* a nosecone. The temperature of the mice was monitored during surgery by a rectal thermometer and maintained at 37°C with a homoeothermic blanket. Abdominal hair was removed by Veet^®^ hair removal cream and the area cleaned with 70% ethanol. The abdomen was opened *via* a 1.5 cm midline incision, exposing the caecum. The caecum was then totally ligated 1.5 cm from the end of the caecum (just below the ileocecal valve) and a G-18 needle was used to puncture both opposite ends of the ligated caecum. A small amount of faeces was squeezed out (~3mm) from both ends and the caecum was placed back into the abdomen in its anatomical position. Thereafter, 5 ml/kg of normal saline (0.9% NaCl) was placed into the abdominal cavity and the abdomen was closed. Normal saline (10 ml/kg) was also given s.c. directly after surgery for fluid resuscitation and this was repeated at both 6 h and 18 h after surgery. Antibiotics (Imipenem/Cilastin; 0.25mg/ml dissolved in the resuscitation fluid s.c.) and an analgesic (buprenorphine; 0.05 mg/kg body weight i.p.) were also administered at 6 h and 18 h after surgery. At 24 h after CLP, cardiac function was assessed by echocardiography *in vivo.* At 24 h, mice were anaesthetized (terminal anaesthesia) with isoflurane and blood was obtained by cardiac puncture. Mice were then killed by removing the heart. Organs were snap frozen in liquid nitrogen for further analysis.

At 24 h, a clinical score of each mouse that underwent surgery was taken to monitor the health of each mouse. The clinical score was based on the following signs: piloerection, diarrhoea, respiratory distress, tremors, lethargy, and periorbital exudates resulting in a potential, maximal score of 6. Mice with a clinical score of ≤ 3 were considered to have moderate sepsis, while mice with a clinical score of > 3 mice were considered to have severe sepsis.

### Study Design 

Pre-treatment study: Mice were randomised into three groups (sham, control, and drug treatment). Sham and CLP control mice received vehicle *via* oral gavage just before CLP surgery and 8 h after CLP, while drug treatment group received RG100204 *via* oral gavage just before CLP and 8 h after CLP surgery.

Post-treatment study: Mice were randomised into five groups (sham, control and three different drug treatments). Sham and CLP control mice received vehicle *via* oral gavage 1 h and 8 h after CLP surgery. The three different timepoints of the administration of RG100204 *via* oral gavage included: 1 h & 8 h, or 3 h & 8 h, or 3 h & 8 h & 18 h, after CLP surgery.

### Assessment of Cardiac Function 

At 24 h after surgery, cardiac function was assessed using the Vevo 3100 imaging system and MX550D transducer (FujiFilm VisualSonics). Mice were initially anaesthetized using 3% isoflurane together with 1 L/min oxygen in an anaesthesia chamber. Once mice were sedated, they were then transferred to the echocardiography table, where they were allowed to stabilize for at least 10 min and maintained under anaesthesia now using 2% isoflurane together with 1 L/min oxygen for the duration of the procedure *via* nosecone. The limbs of the mice were then taped down onto the metal ECG leads on the platform. The platform was heated to ensure the body temperature of the animals was maintained throughout the procedure (37°C). The temperature was continuously monitored with a rectal thermometer and the heart rate was monitored throughout the procedure from the ECG trace. The fur on the chest of the mice was removed using Veet^®^ hair remover. Echocardiography gel, which was pre-warmed, was then placed on the shaven chest of the mice to allow measurements of the heart to be taken using the MX550D transducer. The following parameters were measured: left ventricular ejection fraction (LVEF), fractional shortening (FS), fractional area change (FAC), cardiac output (CO), stroke volume (SV), mitral valve E/A ratio, myocardial performance index, pulmonary artery VTI and pulmonary artery peak velocity. All data was analyzed offline on VevoLab (FujiFilm VisualSonics).

### Organ and Blood Collection

At the end of the experiment mice were anaesthetized with isoflurane (3%) delivered in oxygen (1 L/min). They were then sacrificed by terminal cardiac puncture and exsanguination (removal of blood) with a G25 needle. Approximately 0.7 ml of blood was collected from each mouse and decanted immediately into 1.3 ml serum gel tubes (Sarstedt, Nümbrecht, Germany). All organs were collected and snap frozen in liquid nitrogen and were stored at -80°C. Blood serum was isolated after 3 min centrifugation at 9900 rpm, before snap-freezing in liquid nitrogen and storing at -80°C. 100 μl of serum samples were then analyzed in a blinded fashion by a commercial veterinary testing laboratory (MRC Harwell Institute, Oxfordshire, UK) to evaluate the biomarkers serum urea, and creatinine for renal dysfunction, serum alanine aminotransferase (ALT), and serum aspartate aminotransferase (AST) for hepatocellular injury, and lactate dehydrogenase (LDH) for general organ injury.

### Western Blot Analysis

Immunoblot analyses of heart and kidney tissue were carried out using semi-quantitative Western blotting, as previously described (26). To assess the degree of phosphorylation of IKKα/β at Ser^176/180^, IĸBα at Ser^32/36^, the expression and nuclear translocation of NF-ĸB p65, the expression of NLRP3, including the cleavage of pro-caspase to active caspase 1. The antibodies used were: rabbit anti-Ser^176/180^-IKKα/β (1:1000, Cell Signalling Technology), rabbit anti-total IKKα/β (1:1000, Cell Signalling Technology), mouse anti-Ser^32/36^-IκBα (1:1000, Cell Signalling Technology), mouse anti-total IĸBα (1:1000, Cell Signalling Technology), rabbit anti-NFĸB p65 (1:1000, Cell Signalling Technology), 1:1000 rabbit anti- NLRP3 inflammasome (AdipoGen) and 1:1000 mouse anti-caspase 1 (p20) (Cell Signalling).

The protein bands were visualized using enhanced chemiluminescence (ECL) detection system. Bio-Rad Image Lab Software 6.0.1 was used to analyze the immunoreactive bands and the results were normalized to sham.

### Myeloperoxidase (MPO) Assay

Tissue was weighed and left on ice (~50 mg for lungs). Samples were homogenized in 500 μl of 20 mM phosphate buffer (pH 7.4) on ice. The homogenate was centrifuged at 13,000 x g at 4°C for 10 min. The supernatant was discarded, and the pellet was resuspended in 0.5 mL of hexadecyltrimethylammonium bromide (HTAB) solution. HTAB is a detergent that releases and solubilizes MPO from neutrophilic granules. Samples were centrifuged at 13,000 x g at 4°C for 10 min and supernatant was collected. The plate reader was set to 37°C. In a 96-well plate, 30 µL of the supernatant were added to each well (in duplicate). 180 µl of peroxide solution [sodium phosphate buffer (80 mM PBS, pH 5.4) with H_2_O_2_ (0.4 mM)] was added. The final concentration of H_2_O_2_ in the plate must be 0.3 mM. The reaction started with the addition of 20 µL of the TMB solution (18.4 mM). The final concentration on the TMB plate was 1.6 mM. The mixture was incubated for 2 min in the plate reader at 37°C, before 5 absorbance readings at 650 nm at 2 min intervals. MPO activity was expressed as optical density (O.D.) at 650nm per mg of protein.

### Statistical Analysis

Dose-responses were calculated in GraphPad Prism 5.0 (GraphPad Software, San Diego, California, USA), after log transformation of compound concentrations and fitting to a standard sigmoidal model, with an assumed Hill slope of -1. All other data were analyzed using GraphPad Prism 7.0. All data in both text and figures are expressed as mean ± standard error of the mean (SEM) of *n* observations, where *n* represents the number of animals studied. Data was then assessed using One-way ANOVA followed by a Bonferroni’s *post-hoc* test, except for **Figures 3C, E**, **8C, E**, where 2-way-ANOVA was performed. P < 0.05 was considered statistically significant.

## Results

### RG100204 *In Vitro* Characterization

**Figure 1:**
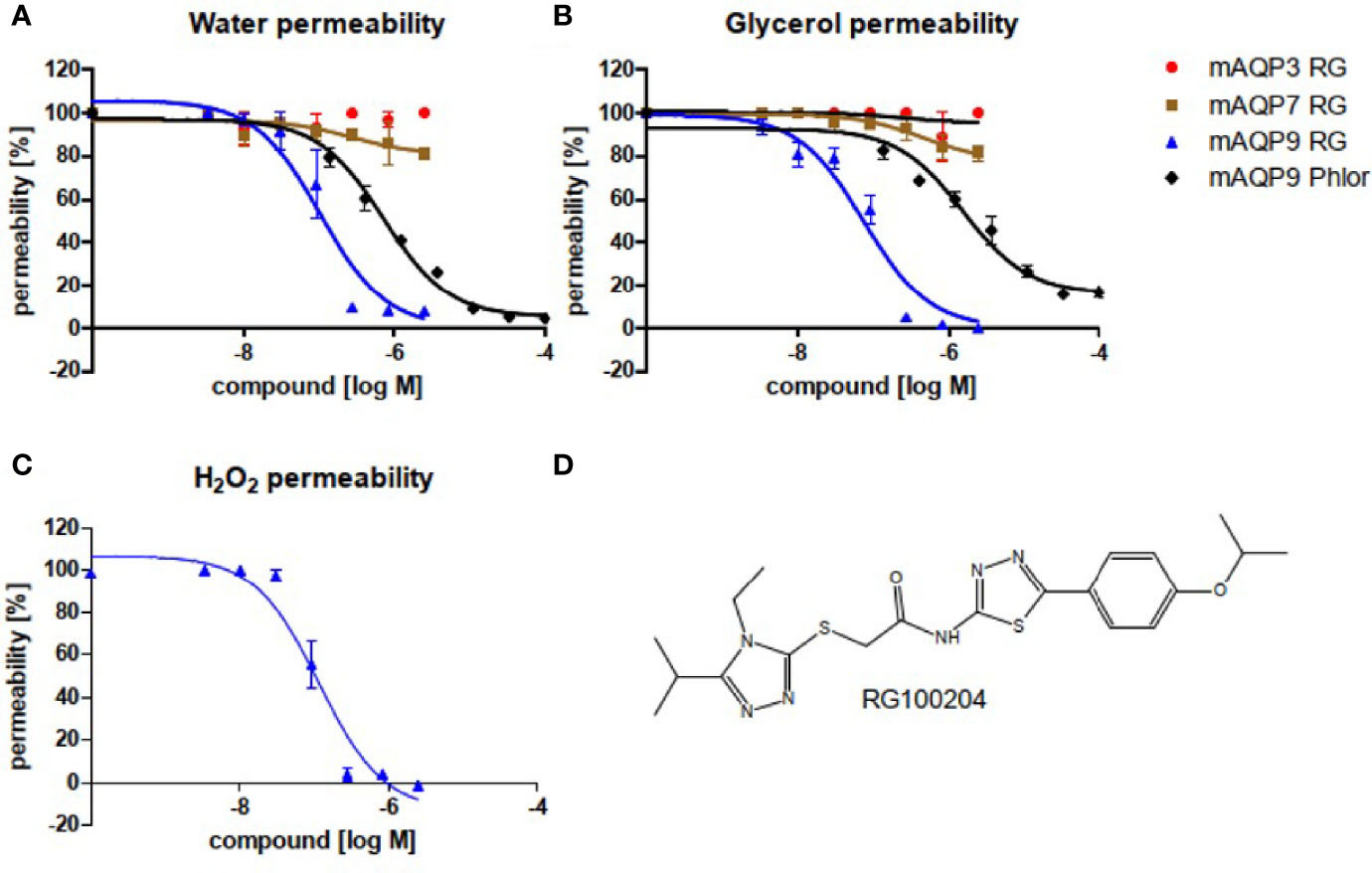
Inhibitor characterization in CHO cells that ectopically express mouse AQP isoforms, as indicated. Water efflux **(A)** and glycerol efflux **(B)** were measured in Calcein loaded cells. H_2_O_2_ permeability was measured in a cell line that expressed mouse AQP9, along with the H_2_O_2_ sensitive fluorescence reporter protein HyPer3 **(C)**. Permeabilities were calculated in comparison to the respective parental cell lines, without AQP integration. **(D)** Chemical structure of RG100204.RG: RG100204, Phlor: Phloretin. Inhibition of water permeability: RG, IC_50_~1.1×10^-7^ M, R^2 =^ 0.91; Phlor IC_50_~7.2×10^-7^ M, R^2 =^ 0.98. Inhibition of glycerol permeability: RG, IC_50_~7.6×10^-8^ M, R^2 =^ 0.94; Phlor IC_50_~1.6×10^-6^ M, R^2 =^ 0.95. Inhibition of H_2_O_2_ permeability: RG, IC_50_~1.2×10^-7^ M, R^2 =^ 0.94. *n *= 3.

In order to characterize RG100204 for AQP isoform inhibition, we tested CHO cells with ectopic expression of either mouse AQP9, or its closest mouse AQP homologues, AQP3, and AQP7, respectively. AQP water permeability and AQP glycerol permeability were tested in Calcein loaded cells, a fluorescent cell volume indicator (**Figures 1A, B**). We observed a dose-dependent inhibition of both, AQP9 water permeability (IC_50_~1.1×10^-7^ M) and AQP9 glycerol permeability (IC_50_~7.6×10^-8^ M) by RG100204. By comparison, inhibition by the known, unspecific AQP9 inhibitor phloretin was less potent (IC_50_~7.2×10^-7^ M), and for glycerol permeability also less efficacious (IC_50_~1.6×10^-6^ M, mean observed remaining permeability 16%). No inhibition of water or glycerol permeability by RG100204 was observed in AQP3 expressing CHO cells, while only minor inhibition of AQP7 dependent water and glycerol permeability could be measured, thus establishing that RG100204 inhibits AQP9, while it does not inhibit closely related AQP isoforms.

In order to establish that RG100204 can inhibit AQP9 H_2_O_2_ permeability we utilized a cell line that expresses mouse AQP9 along with the H_2_O_2_ specific sensor HyPer-3. We observed inhibition of AQP9 H_2_O_2_ permeability by RG100204 with similar potency and efficacy as for water and glycerol permeability (**Figure 1C**).

**Figure 2:**
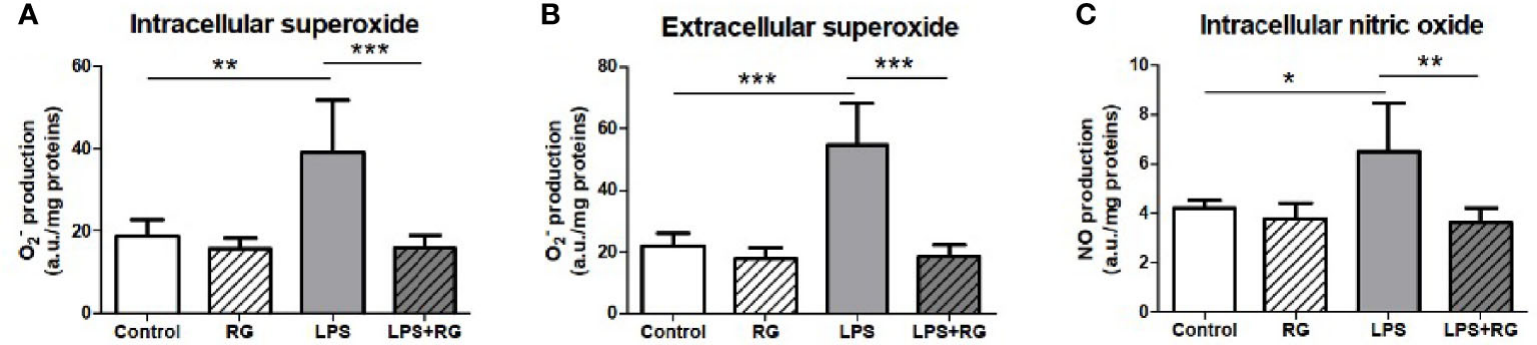
LPS treatment of FaO rat hepatoma cells resulted in increased superoxide concentration, both intracellularly **(A)**, and extracellularly **(B)**, as well as increased intracellular nitric oxide concentration **(C)**. Oxide increases were diminished by RG100204 treatment. The levels of ${\text{O}}_{2}^{-}$ and NO were assayed by electron paramagnetic resonance of spin traps prepared from the FaO cells submitted to the different experimental conditions. Control, cells exposed to the vehicle (1% DMSO) alone; RG, 25 μM RG100204 in 1% DMSO; LPS, 1 µg LPS/mL in 1% DMSO; LPS+RG, 25 µM RG100204 plus 1 µg LPS/mL in 1% DMSO; a.u., arbitrary units. Data were analyzed by one-way ANOVA, with Bonferroni’s *post-hoc* test. Data are expressed as mean ± SEM, n=5, *P < 0.05, **P < 0.01 and ***P < 0.001.

To further establish that RG100204 affected similar biological processes as was established previously for *Aqp9* gene deletion, we next evaluated the effect of RG100204 on LPS-induced NO and ${\text{O}}_{2}^{-}$ production in FaO cells, a rat hepatoma cell line expressing AQP9 (27). LPS increased the concentration of extracellular and intracellular ${\text{O}}_{2}^{-}$ and intracellular NO in FaO cells compared to vehicle treated controls. This LPS induced increase in free radicals was completely prevented in cells incubated with RG100204 (**Figure 2**). At the dose used for the *in vitro* characterization RG100204 or LPS alone or RG100204 plus LPS did not show any significant toxic effect on FaO cells viability (**Supplementary Figure 1**).

### RG100204 Treatment of Septic Shock in Mice

In order to address if RG100204 can affect systemic inflammation *in vivo* we utilized a CLP mouse model of polymicrobial sepsis resulting from intestinal perforation. The model includes fluid resuscitation and antibiotics treatment, thus mimicking the clinical standard of care in bacterial sepsis.

### Pre-Treatment With RG100204 Maintains Physiological Parameters After CLP Surgery

**Figure 3:**
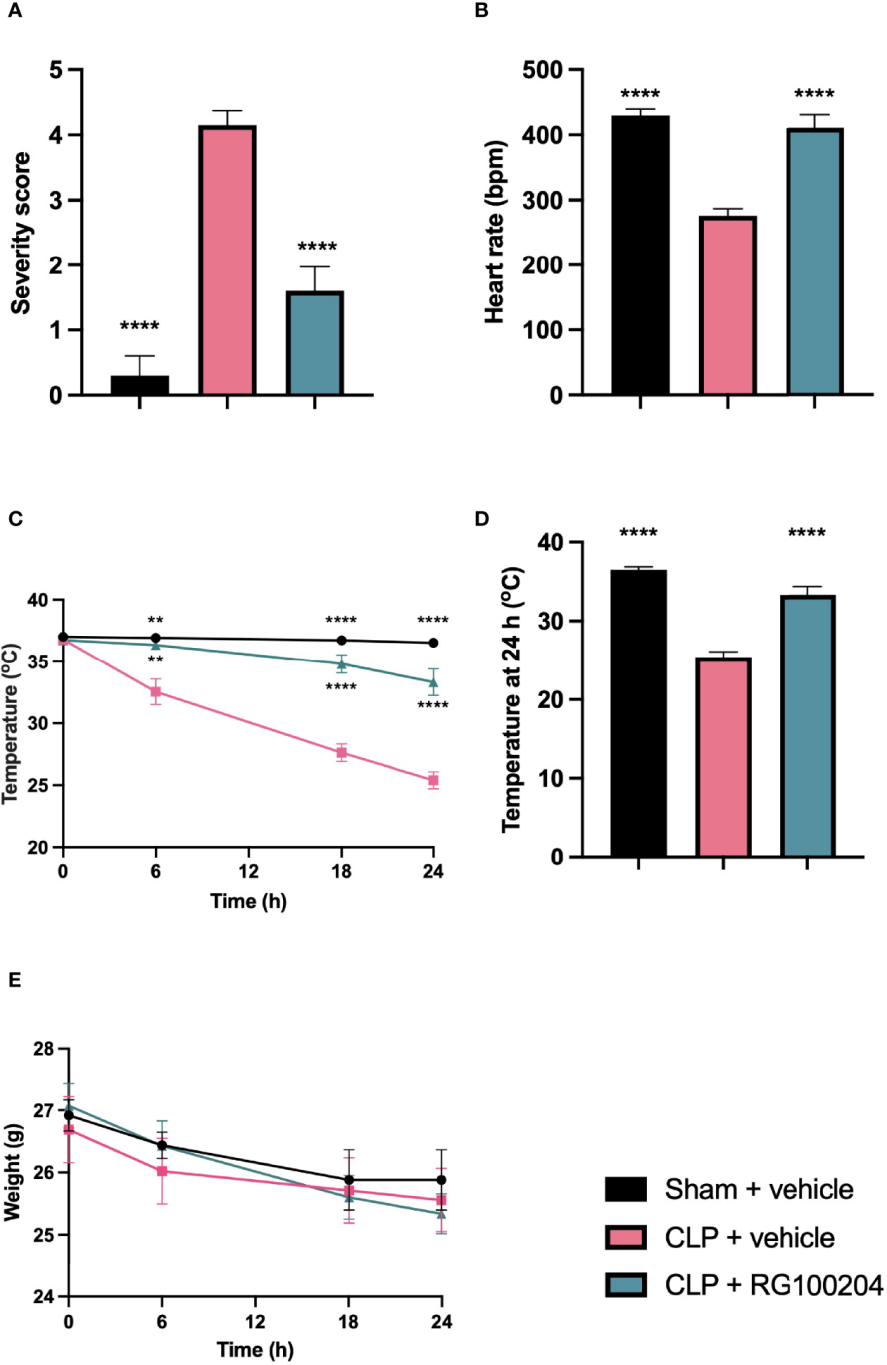
Effect of pre-treatment with RG100204 on physiological parameters 24 h after CLP surgery. Mice were pre-treated with vehicle or RG100204 before CLP and 8 h after CLP surgery. Over the 24 h period after CLP physiological parameters were measured **(A)** Severity score at 24 h; **(B)** heart rate at 24 h (bpm); **(C)** temperature (°C); **(D)** temperature at 24 h (°C); and **(E)** weight (g). The following groups were studied: sham + vehicle (n = 5), CLP + vehicle (n = 10) and CLP + RG100204 (n = 10). All data were analyzed by one-way and two-way ANOVA, followed by a Bonferroni’s *post-hoc* test. Data are expressed as mean ± SEM. **P < 0.01 and ****P < 0.0001 vs. CLP + vehicle.

When compared to sham-operated mice, mice subjected to CLP and treated with vehicle showed a significant increase in severity score and a decrease in heart rate after 24 h (**Figures 3A, B**). The temperature was also significantly decreased in this group of animals at 6 h, 18 h and 24 h (**Figure 3C**). When compared to CLP-animals treated with vehicle, treatment of CLP-animals with RG100204 just before CLP and 8 h after CLP surgery significantly attenuated the severity score and the decline in temperature at 6 h, 18 h and 24 h after CLP (**Figures 3A–D**). All mice showed a decline in body weight over the 24 h observation period, but no significant difference was detected between any of the three groups (sham + vehicle, CLP + vehicle and CLP + RG100204) (**Figure 3E**).

### Pre-Treatment With RG100204 Attenuates CLP-Induced Systolic Cardiac Dysfunction

**Figure 4:**
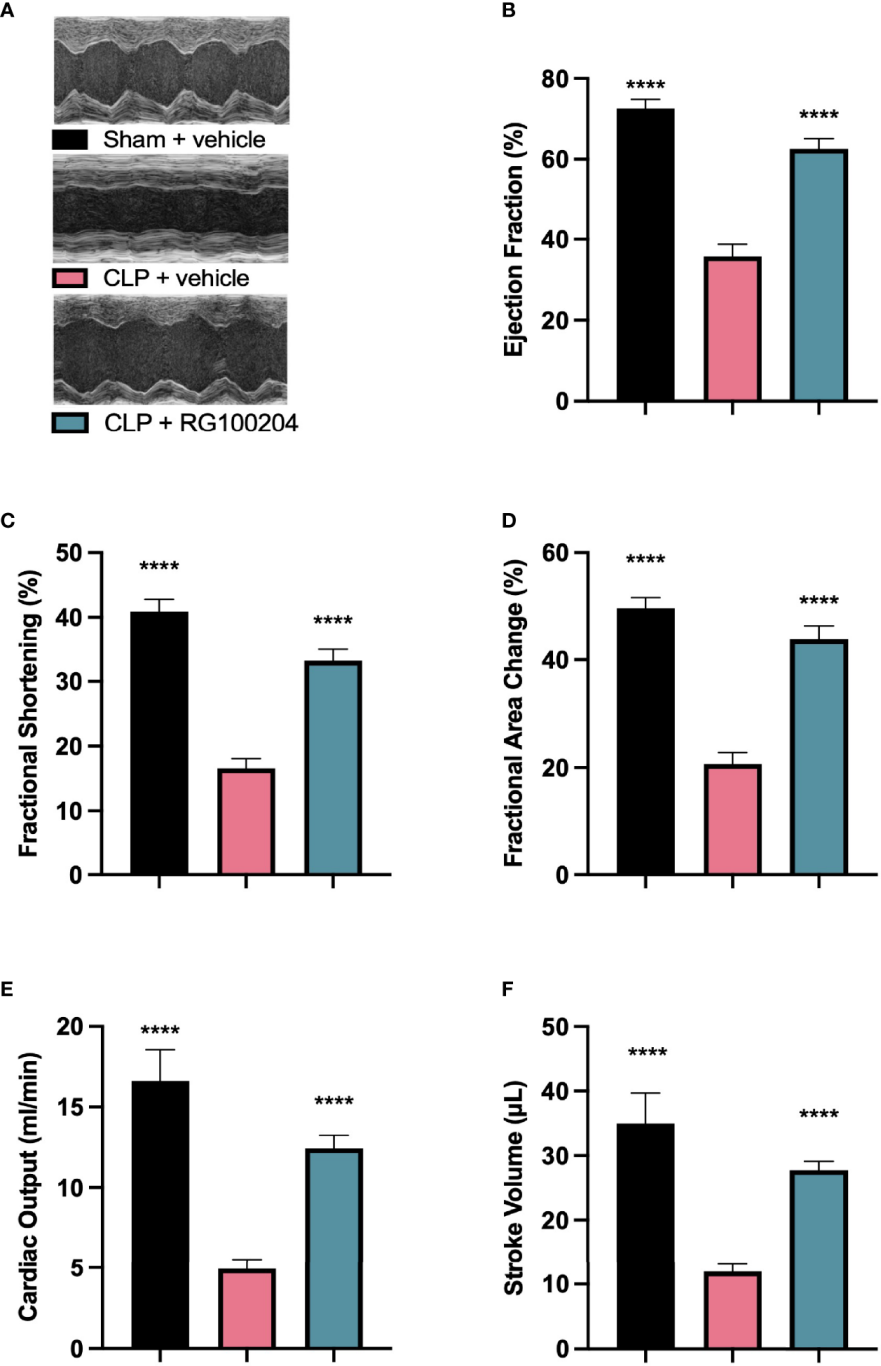
Effect of pre-treatment with RG100204 on CLP-induced systolic cardiac dysfunction. Mice were pre-treated with vehicle or RG100204 before CLP and 8 h after CLP. Systolic cardiac function was assessed after 24 h. **(A)** Representative M-mode echocardiogram; **(B)** Ejection fraction (%); **(C)** Fractional shortening (%); **(D)** Fractional area change (%); **(E)** Cardiac output (ml/min); and **(F)** Stroke volume (μL). The following groups were studied: sham + vehicle (n = 5), CLP + vehicle (n = 10) and CLP + RG100204 (n = 10). All data were analyzed by one-way ANOVA, followed by a Bonferroni’s *post-hoc* test. Data are expressed as mean ± SEM. ****P < 0.0001 vs. CLP + vehicle.

When compared to sham-operated mice, mice subjected to CLP and treated with vehicle demonstrated a significant reduction in the systolic parameters measured: % EF, % FS, % FAC, CO and SV after 24 h, indicating the development of systolic, cardiac dysfunction (**Figures 4A–F**). When compared to CLP-animals treated with vehicle, treatment of CLP-animals with RG100204 just before CLP and 8 h after CLP surgery improved systolic cardiac dysfunction with a significant increase in the following cardiac parameters: % EF, % FS, % FAC, CO and SV, demonstrating the ability of pre-treatment with RG100204 to effectively attenuate systolic dysfunction in septic mice (**Figures 4A–F**).

### Pre-Treatment With RG100204 Attenuates CLP-Induced Diastolic Cardiac Dysfunction

**Figure 5:**
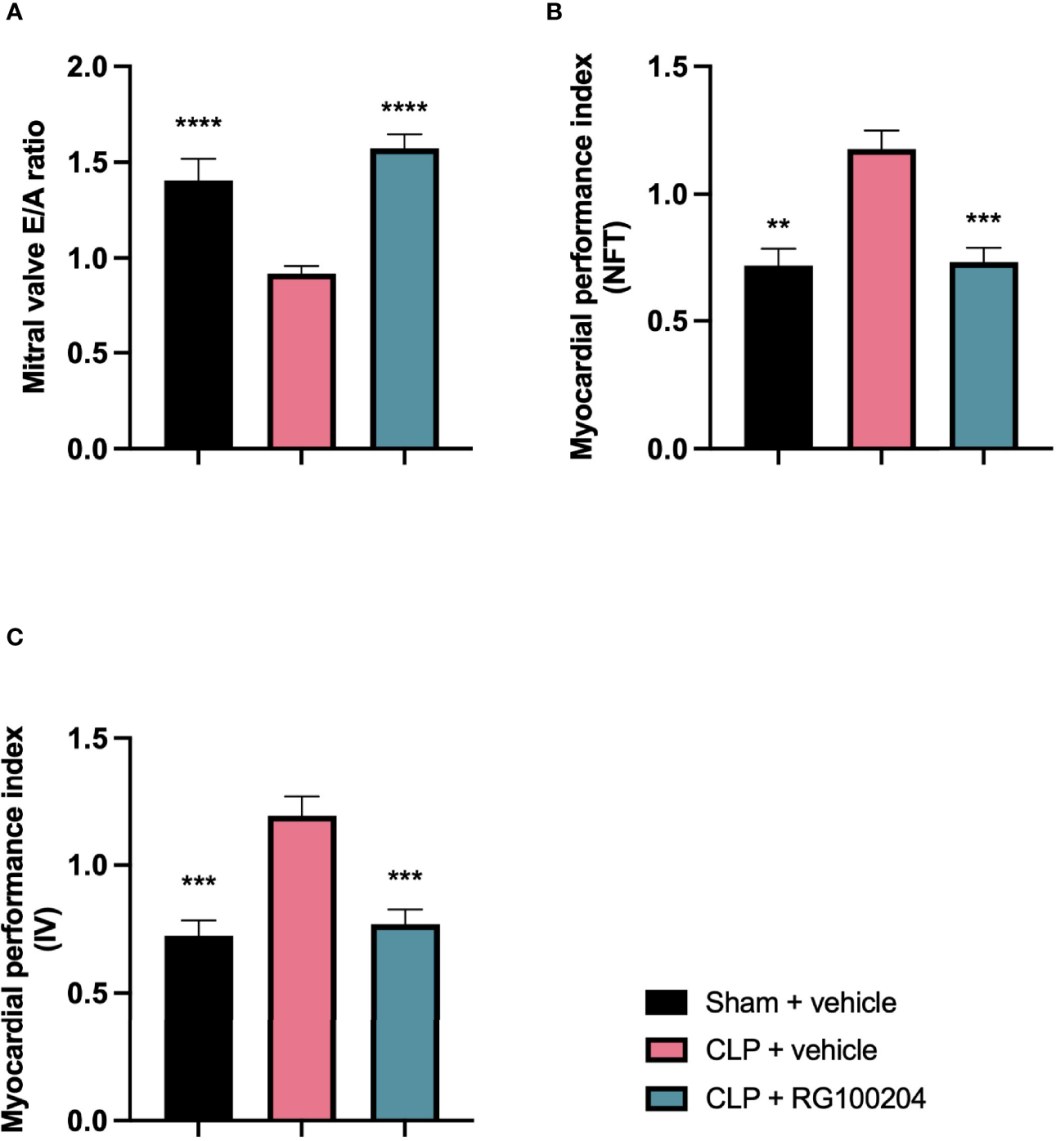
Effect of pre-treatment with RG100204 on CLP-induced diastolic cardiac dysfunction. Mice were pre-treated with vehicle or RG100204 just before CLP and 8 h after CLP. Diastolic cardiac function was assessed after 24 h. **(A)** Mitral valve E/A ratio; **(B)** Myocardial performance index (NFT); and **(C)** Myocardial performance index (IV). The following groups were studied: sham + vehicle (n = 5), CLP + vehicle (n = 10) and CLP + RG100204 (n = 10). All data were analyzed by one-way ANOVA, followed by a Bonferroni’s *post-hoc* test. Data are expressed as mean ± SEM. **P < 0.01, ***P < 0.001, and ****P < 0.0001 vs. CLP + vehicle.

When compared to sham-operated mice, mice subjected to CLP for 24 h and treated with vehicle demonstrated a significant reduction in the mitral valve E/A ratio (**Figure 5A**) and a significant increase in the myocardial performance index NFT and IV (**Figures 5B, C**) indicating the development of diastolic cardiac dysfunction. When compared to CLP-animals treated with vehicle, CLP-animals treated with RG100204 just before and 8 h after CLP significantly attenuated the decline in the mitral valve E/A ratio and the rise of the myocardial performance index NFT and IV (**Figures 5A–C**) and, hence, the diastolic, cardiac dysfunction caused by CLP.

### Pre-Treatment With RG100204 Maintains Pulmonary Artery Flow

**Figure 6:**
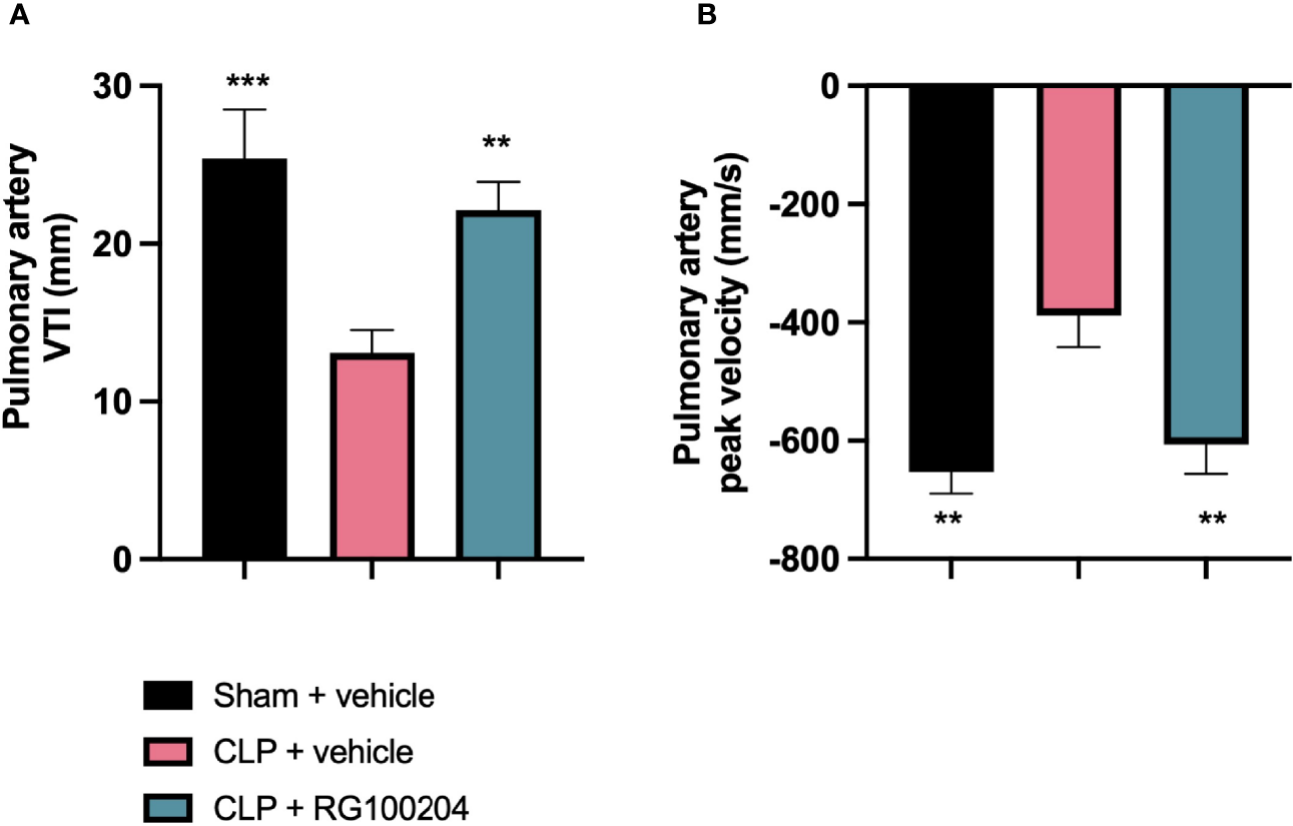
Pre-treatment with RG100204 maintains pulmonary artery flow. Mice were pre-treated with vehicle or RG100204 before CLP and 8 h after CLP. Pulmonary artery flow was assessed after 24 h. **(A)** Pulmonary artery VTI; **(B)** Pulmonary artery peak velocity. The following groups were studied: sham + vehicle (n = 5), CLP + vehicle (n = 10) and CLP + RG100204 (n = 10). All data were analyzed by one-way ANOVA, followed by a Bonferroni’s *post-hoc* test. Data are expressed as mean ± SEM. **P < 0.01 and ***P < 0.001 vs. CLP + vehicle.

When compared to sham-operated mice, mice subjected to CLP for 24 h and treated with vehicle demonstrated a significant reduction in the pulmonary artery velocity time integral VTI and peak velocity (**Figures 6A, B**). However, CLP-animals treated with RG100204 just before CLP and 8 h after CLP, significantly attenuated the reduction in pulmonary artery VTI and peak velocity caused by CLP (**Figures 6A, B**).

### Pre-Treatment With RG100204 Attenuates CLP-Induced Hepatocellular Injury and Renal Dysfunction

**Figure 7:**
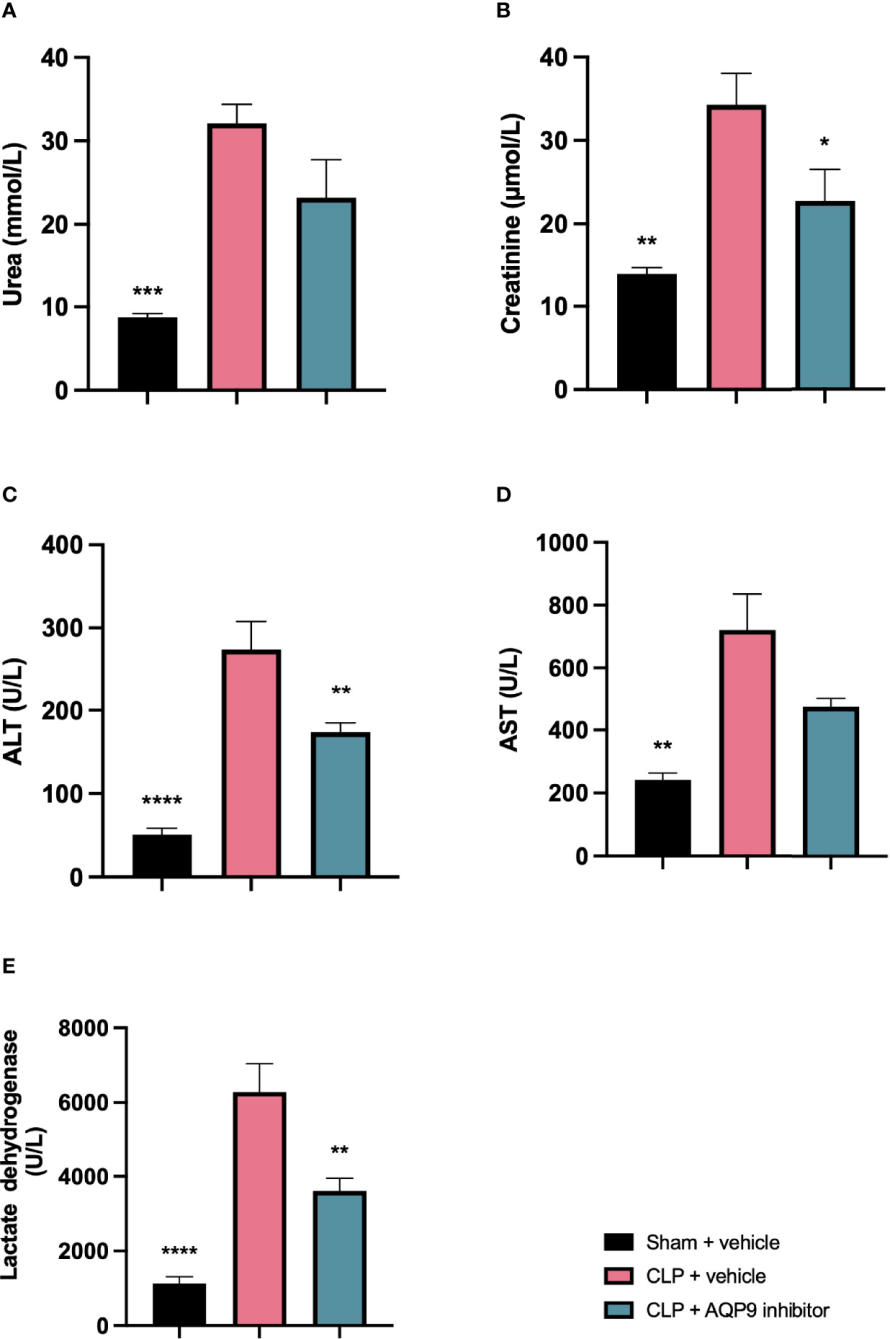
Pre-treatment with RG100204 attenuates CLP-induced hepatocellular injury and renal dysfunction. Mice were pre-treated with vehicle or RG100204 just before CLP and 8 h after CLP. At 24 h after CLP surgery blood samples were collected to analyze. **(A)** Urea (mmol/L); **(B)** Creatinine (μmol/L); **(C)** Alanine aminotransferase (ALT) (U/L); **(D)** Aspartate transaminase (AST) (U/L); and **(E)** Lactate dehydrogenase (U/L). The following groups were studied: sham + vehicle (n = 5), CLP + vehicle (n = 10) and CLP + RG100204 (n = 10). All data were analyzed by one-way ANOVA, followed by a Bonferroni’s *post-hoc* test. Data are expressed as mean ± SEM. *P < 0.05, **P < 0.01, ***P < 0.001, and ****P < 0.0001 vs. CLP + vehicle.

When compared to sham-operated mice, mice subjected to CLP for 24 h and treated with vehicle developed kidney dysfunction (indicated by a significant rise in serum urea and creatinine) and hepatocellular injury (indicated by a significant increase in serum ALT and AST). CLP-sepsis also caused a rise in the cell injury marker LDH (**Figures 7A–E**). When compared to CLP-animals treated with vehicle, CLP-animals treated with RG100204 just before CLP and 8 h after CLP showed significant reduction in serum creatinine, LDH and ALT, but no significant reductions in serum urea or AST (**Figures 7A–E**).

### Effect of Post-Treatment (Therapeutic Administration) With RG100204 on Physiological Parameters After CLP

**Figure 8:**
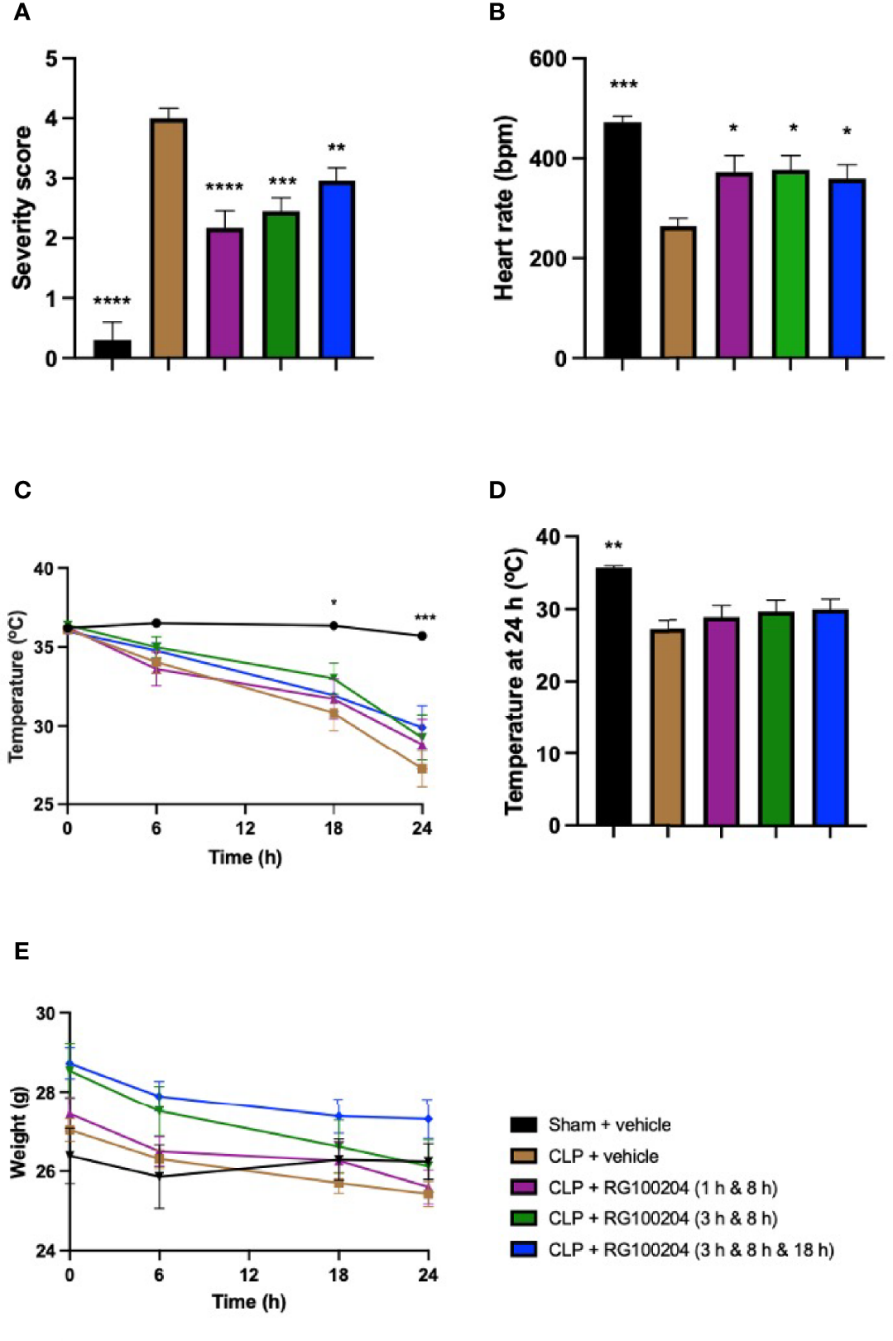
Effect of post-treatment (therapeutic administration) with RG100204 on physiological parameters after CLP. Mice received post-treatment with vehicle or RG100204 at different dosing times after CLP. Over the 24 h period after CLP physiological parameters were measured. **(A)** Severity score; **(B)** heart rate at 24 h (bpm); **(C)** temperature (°C); **(D)** temperature at 24 h (°C); and **(E)** weight at 24 h (g). The following groups were studied: sham + vehicle (n = 5), CLP + vehicle (n = 10), CLP + RG100204 (1 h & 8 h) (n = 10), CLP + RG100204 (3 h & 8 h) (n = 10) and CLP + RG100204 (3 h & 8 h & 18 h) (n = 10). All data is expressed as mean ± SEM for n number of observations. All data were analyzed by one-way and two-way ANOVA, followed by a Bonferroni’s *post-hoc* test. Data are expressed as mean ± SEM. *P < 0.05, **P < 0.01, ***P < 0.001, and ****P < 0.0001 vs. CLP + vehicle.

When compared to sham-operated mice, mice subjected to CLP and treated with vehicle showed a significant increase in severity score and a decrease in heart rate after 24 h (**Figures 8A, B**). The temperature was also significantly decreased in these animals at 6 h, 18 h and 24 h (**Figure 8C**). When compared to CLP-animals treated with vehicle, treatment of CLP-animals with RG100204 at all dosing times significantly attenuated the severity score and the rise in heart rate but did not have a significant effect on hypothermia (**Figures 8A–D**). Over the 24 h period, the body weight of all mice declined, but no significant differences were detected between any of the groups studied (**Figure 8E**).

### Effect of Post-Treatment (Therapeutic Administration) With RG100204 on CLP-Induced Systolic, Cardiac Dysfunction

**Figure 9:**
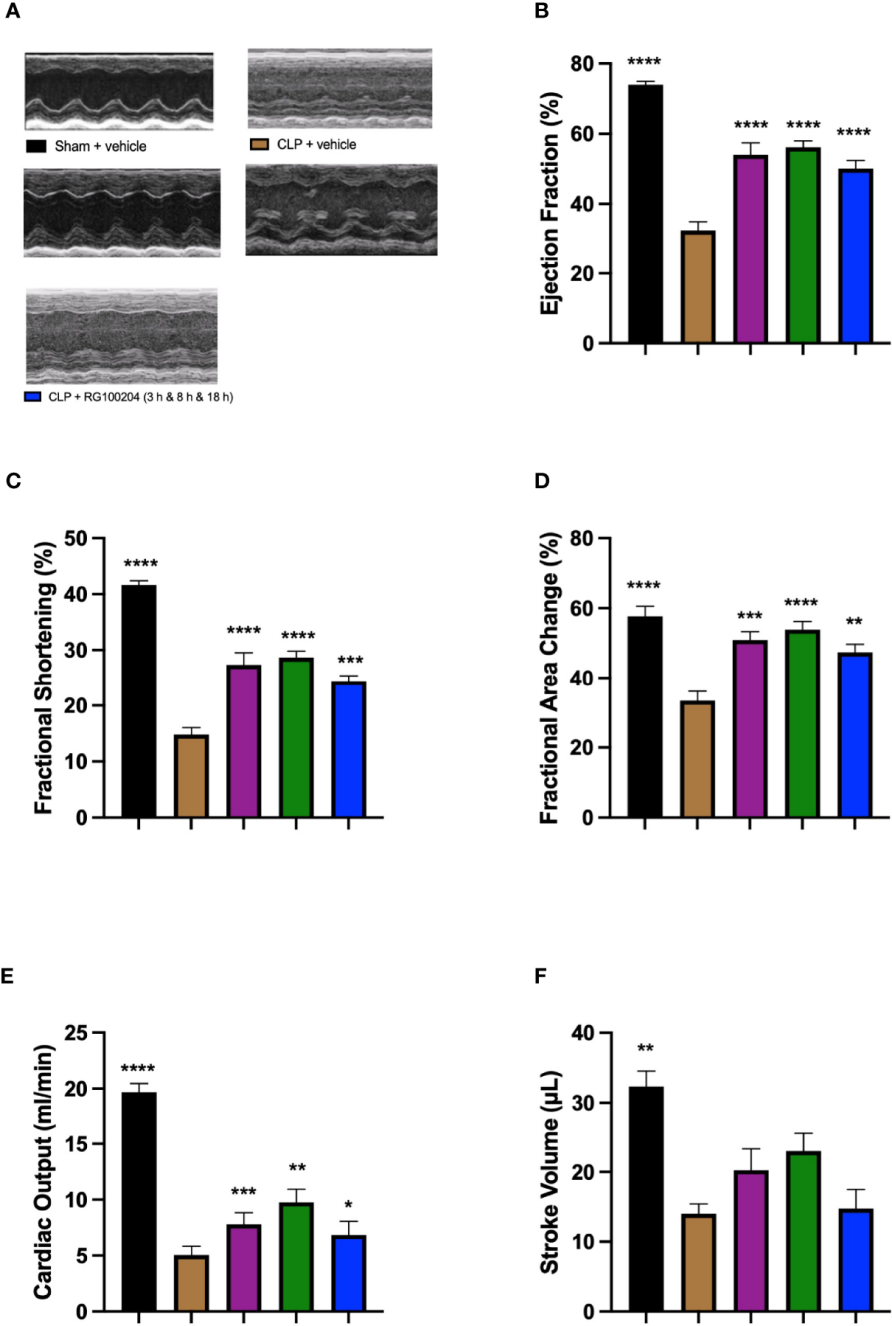
Effect of post-treatment (therapeutic administration) with RG100204 on CLP-induced systolic, cardiac dysfunction. Mice received a post-treatment with vehicle or RG100204 at different dosing times after CLP. Cardiac function was assessed after 24 h. **(A)** Representative M-mode echocardiogram; **(B)** Ejection fraction (%); **(C)** Fractional shortening (%); **(D)** Fractional area change (%); **(E)** Cardiac output (ml/min); and **(F)** Stroke volume (μL). The following groups were studied: sham + vehicle (n = 5), CLP + vehicle (n = 10), CLP + RG100204 (1 h & 8 h) (n = 10), CLP + RG100204 (3 h & 8 h) (n = 10) and CLP + RG100204 (3 h & 8 h & 18 h) (n = 10). All data is expressed as mean ± SEM for n number of observations. All data were analyzed by one-way ANOVA, followed by a Bonferroni’s *post-hoc test*. Data are expressed as mean ± SEM. *P < 0.05, **P < 0.01, ***P < 0.001, and ****P < 0.0001 vs. CLP + vehicle.

When compared to sham-operated mice, mice subjected to CLP for 24 h and treated with vehicle (control) demonstrated a significant reduction in % EF, % FS, % FAC, CO and SV (**Figures 9A–F**), indicating the development of systolic, cardiac dysfunction. When compared to CLP-animals treated with vehicle, treatment of CLP-animals with RG100204 significantly attenuated the decline in % EF, % FS, % FAC and CO caused by CLP except for the latest dosing time (**Figures 9A–F**).

### Effect of Post-Treatment (Therapeutic Administration) With RG100204 on CLP-Induced Diastolic Cardiac Dysfunction

**Figure 10:**
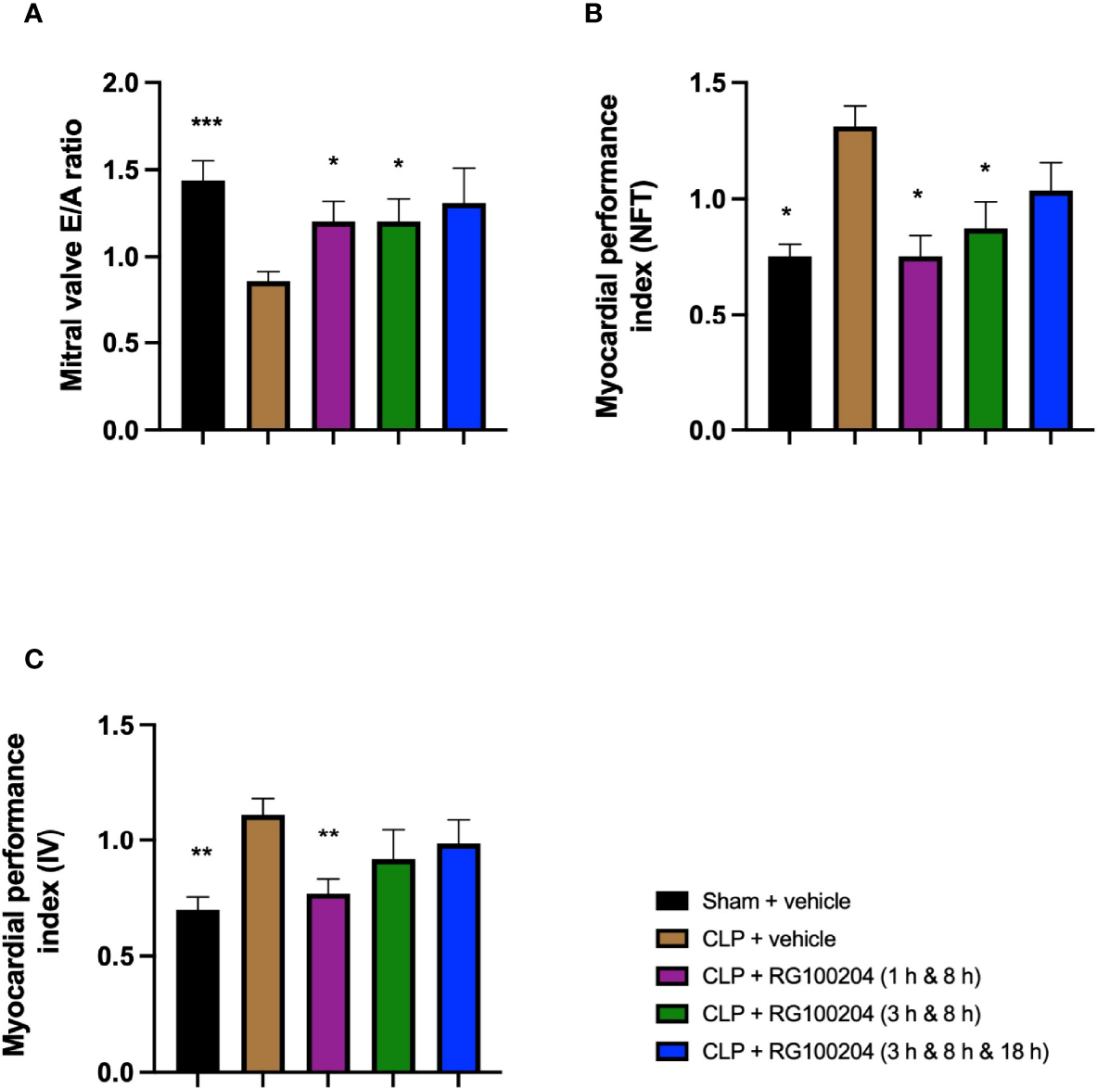
Effect of post-treatment (therapeutic administration) with RG100204 on CLP-induced diastolic cardiac dysfunction. Mice received a post-treatment with vehicle or RG100204 at different dosing times after CLP. Cardiac function was assessed after 24 h. **(A)** Mitral valve E/A ratio; **(B)** Myocardial performance index (NFT); and **(C)** Myocardial performance index (IV). The following groups were studied: sham + vehicle (n = 5), CLP + vehicle (n = 10), CLP + RG100204 (1 h & 8 h) (n = 10), CLP + RG100204 (3 h & 8 h) (n = 10) and CLP + RG100204 (3 h & 8 h & 18 h) (n = 10). All data are expressed as mean ± SEM for n number of observations. All data were analyzed by one-way ANOVA, followed by a Bonferroni’s *post-hoc* test. Data are expressed as mean ± SEM. *P < 0.05, **P < 0.01 and ***P < 0.001 vs. CLP + vehicle.

When compared to sham-operated mice, mice subjected to CLP for 24 h and treated with vehicle (control) demonstrated a significant reduction in the mitral valve E/A ratio (**Figure 10A**) and a significant increase in the myocardial performance index NFT and IV (**Figures 10B, C**) indicating the development of diastolic, cardiac dysfunction. When compared to CLP-animals treated with vehicle, treatment of CLP-animals with RG100204 (except at the latest dosing time) significantly attenuated the decline in the mitral valve E/A ratio and the rise of the myocardial performance index (**Figures 10A, B**) and, hence, the diastolic, cardiac dysfunction caused by CLP.

### Effect of Post-Treatment (Therapeutic Administration) With RG100204 on Pulmonary Artery Flow

**Figure 11:**
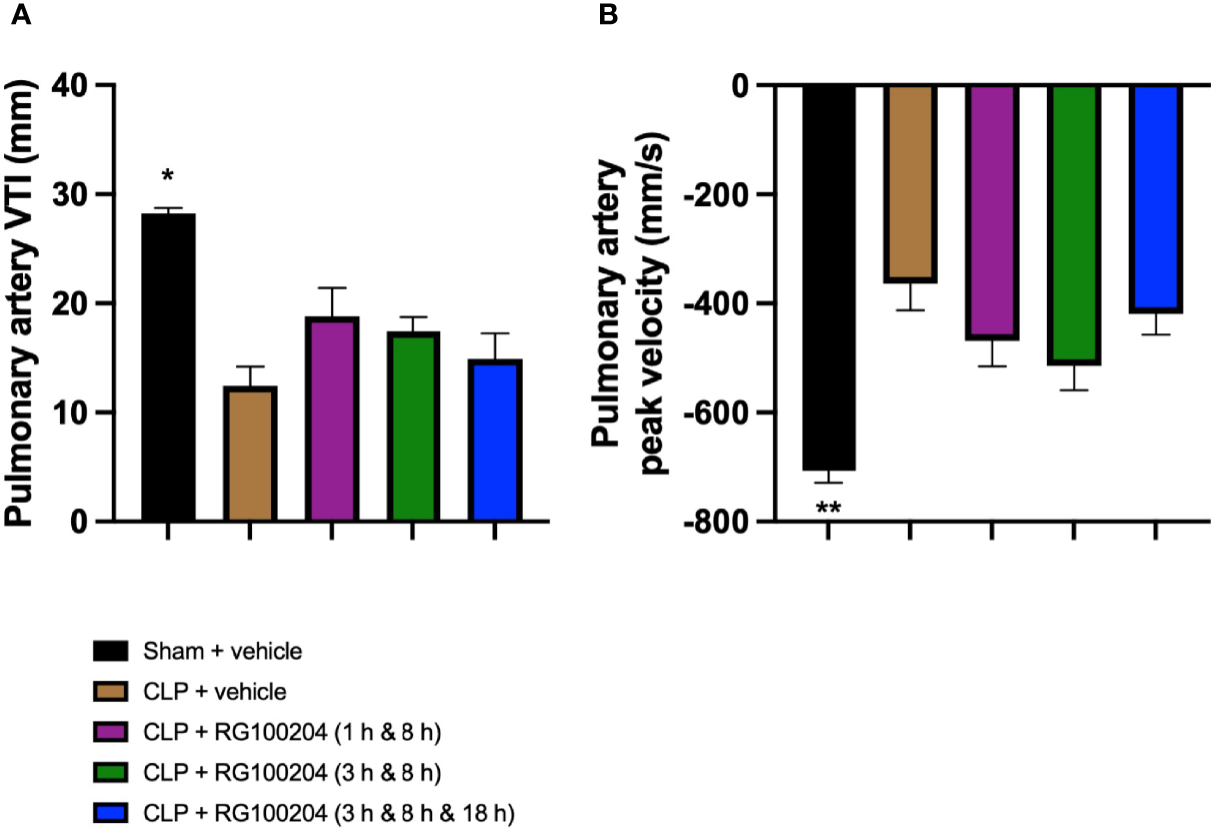
Effect of post-treatment (therapeutic administration) with RG100204 on pulmonary artery flow. Mice received a post-treatment with vehicle or RG100204 at different dosing times after CLP. Cardiac function was assessed after 24 h. **(A)** Pulmonary artery VTI (mm); and **(B)** Pulmonary artery peak velocity (mm/s). The following groups were studied: sham + vehicle (n = 5), CLP + vehicle (n = 10), CLP + RG100204 (1 h & 8 h) (n = 10), CLP + RG100204 (3 h & 8 h) (n = 10) and CLP + RG100204 (3 h & 8 h & 18 h) (n = 10). All data are expressed as mean ± SEM for n number of observations. All data were analyzed by one-way ANOVA, followed by a Bonferroni’s *post-hoc* test. Data are expressed as mean ± SEM. *P < 0.05 and **P < 0.01 vs. CLP + vehicle.

When compared to sham-operated mice, mice subjected to CLP for 24 h and treated with vehicle (control) demonstrated a significant reduction in the pulmonary artery velocity time integral (VTI) and peak velocity (**Figures 11A, B**). When compared to CLP-animals treated with vehicle, CLP-animals treated with RG100204 showed a small but not significant increase in pulmonary artery VTI and peak velocity caused by CLP, with treatment at 1 h & 8 h, and 3 h & 8 h being more effective (**Figures 11A, B**).

### Effect of Post-Treatment (Therapeutic Administration) With RG100204 on CLP-Induced Hepatocellular Injury and Renal Dysfunction

**Figure 12:**
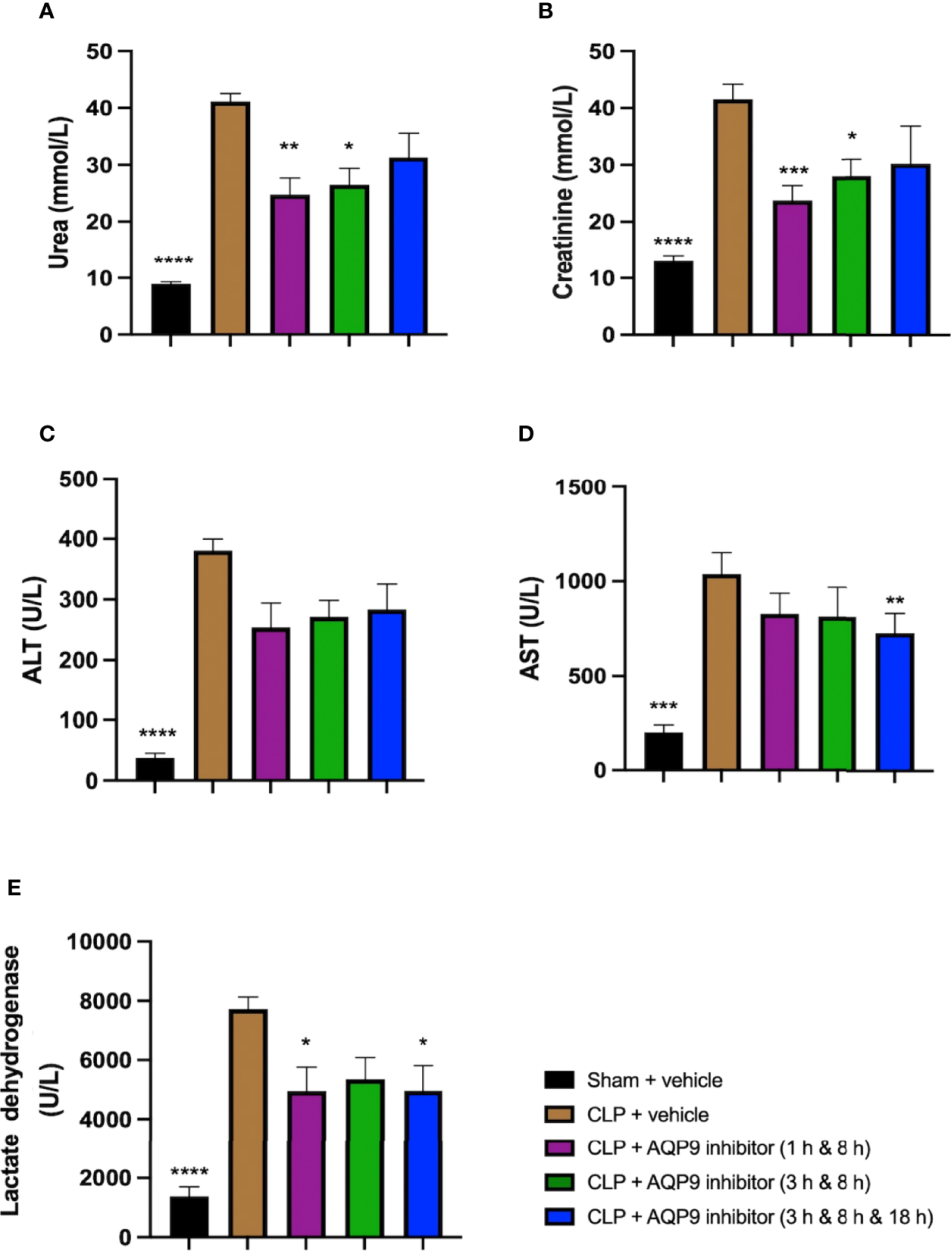
Effect of post-treatment (therapeutic administration) with RG100204 on CLP-induced hepatocellular injury and renal dysfunction. Mice received a post-treatment with vehicle or RG100204 at different dosing times after CLP. Cardiac function was assessed after 24 h. **(A)** Urea (mmol/L); **(B)** Creatinine (μmol/L); **(C)** Alanine aminotransferase (ALT) (U/L); **(D)** Aspartate transaminase (AST) (U/L); and **(E)** Lactate dehydrogenase (U/L). The following groups were studied: sham + vehicle (n = 5), CLP + vehicle (n = 10), CLP + RG100204 (1 h & 8 h) (n = 10), CLP + RG100204 (3 h & 8 h) (n = 10) and CLP + RG100204 (3 h & 8 h & 18 h) (n = 10). All data are expressed as mean ± SEM for n number of observations. All data were analyzed by one-way ANOVA, followed by a Bonferroni’s *post-hoc* test. Data are expressed as mean ± SEM. *P < 0.05, **P < 0.01, ***P < 0.001, and ****P < 0.0001 vs. CLP + vehicle.

When compared to sham-operated mice, mice subjected to CLP for 24 h and treated with vehicle (control) developed both kidney dysfunction (rise in urea and creatinine) and hepatocellular injury (rise in ALT and AST) and a rise in the cell injury marker LDH (**Figures 12A–E**). When compared to CLP-animals treated with vehicle, CLP-animals treated with RG100204 at all dose times showed a decrease (although not significant in all) in serum urea, creatinine, LDH, ALT and AST (**Figures 12A–E**). Thus, post-treatment of CLP-mice with RG100204 had a more pronounced effect on renal dysfunction than on hepatocellular injury, caused by CLP.

### Effect of Post-Treatment (Therapeutic Administration) With RG100204 on NF-ĸB Signalling and NLRP3 Inflammasome Activation in the Heart

**Figure 13:**
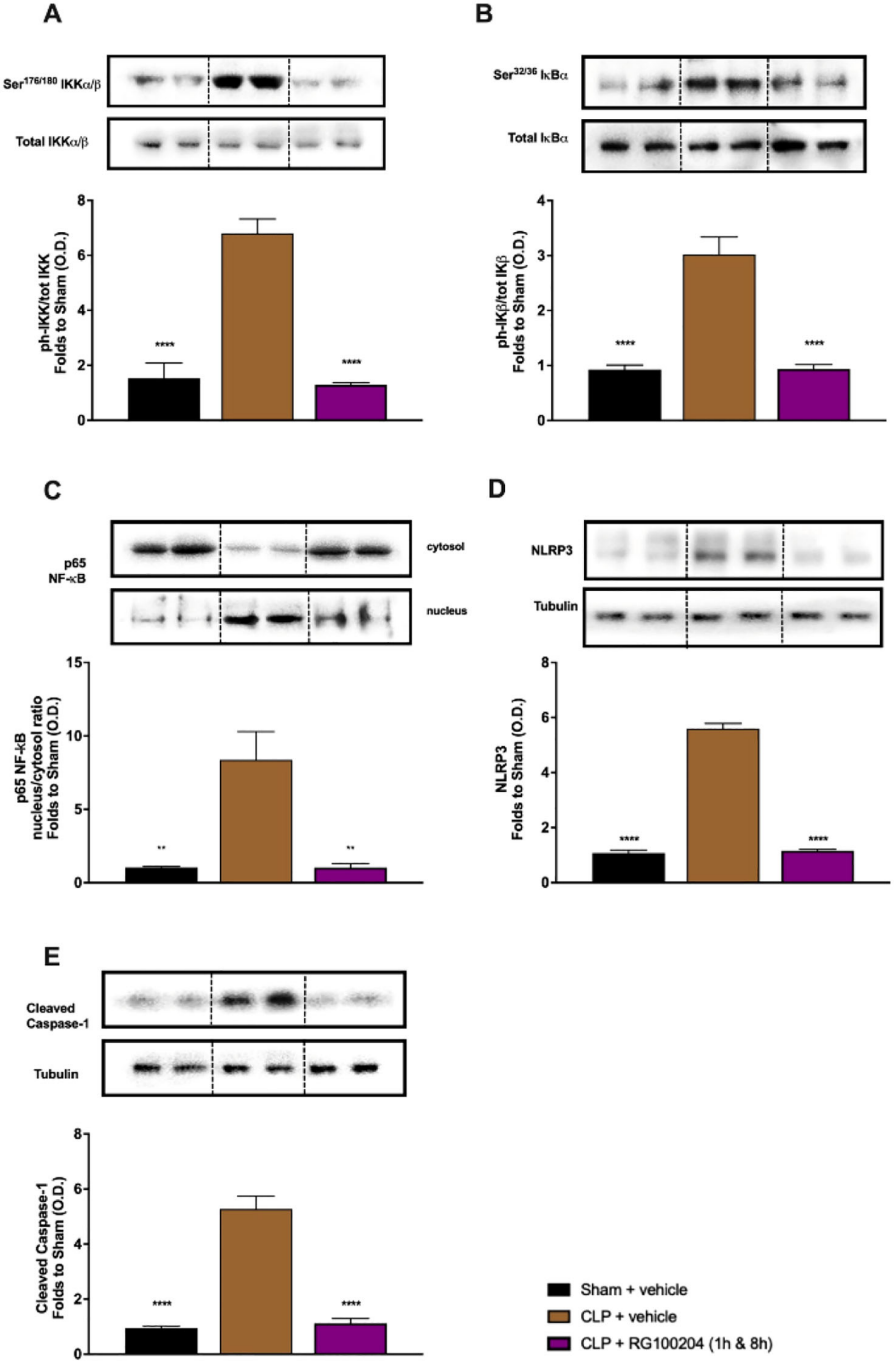
Effect of post-treatment (therapeutic administration) with RG100204 on the NF-ĸB signalling pathway and the activation NLRP3 inflammasome in the heart. Heart samples were collected at the end of the experiment and the NF-κB signalling pathway, as well as the activation of the NLRP3 inflammasome. Densitometry analysis of the bands is expressed as relative optical density (O.D.) of the **(A)** phosphorylation of IKKα/β at Ser^178/180^ corrected for the corresponding total IKKα/β content and normalized using the related sham band; **(B)** phosphorylation of IĸBα at Ser^32/36^ corrected for the corresponding total IĸBα content and normalized using the related sham band; **(C)** NF-ĸB p65 subunit levels in both, cytosolic and nuclear fractions expressed as a nucleus/cytosol ratio normalized using the related sham bands; **(D)** NLRP3 activation, corrected against tubulin and normalized using the related sham bands; and **(E)** proteolytic cleavage of pro-caspase-1 to activated caspase-1 and normalized using the related sham band. The following groups were studied: sham + vehicle (n = 5), CLP + vehicle (n = 10), CLP + RG100204 (1 h & 8 h) (n = 10). All data were analyzed by one-way ANOVA, followed by a Bonferroni’s *post-hoc* test. Data are expressed as mean ± SEM. **P < 0.01 and ****P < 0.0001 vs. the respective sham-operated group.

When compared to sham-operated mice, mice subjected to CLP and treated with vehicle (control) showed significant increases in the phosphorylation of IKKα/β at Ser^176/180^, the phosphorylation of IĸBα at Ser^32/36^ and the translocation of NF-ĸB subunit p65 to the nucleus in the heart (**Figures 13A–C**). When compared to CLP-animals treated with vehicle, treatment of CLP-animals with RG100204 at 1 h followed by 8 h after CLP significantly attenuated the degree of phosphorylation of IKKα/β at Ser^176/180^, the phosphorylation of IĸBα at Ser^32/36^ and the translocation of the p65 subunit of NF-ĸB to the nucleus in the heart (**Figures 13A–C**), indicating the ability of RG100204 to block NF-ĸB activation in the heart. When compared to sham-operated mice, mice subjected to CLP and treated with vehicle (control) demonstrated a significant increase in the expression of the NLRP3 inflammasome and the cleavage of pro-caspase-1 to caspase-1 in the heart (**Figures 13D, E**). However, the expression of the NLPR3 inflammasome was significantly attenuated by the treatment with RG100204 given 1 h followed by 8 h after CLP surgery (**Figures 13D, E**).

### Effect of Post-Treatment (Therapeutic Administration) With RG100204 on NF-ĸB Signalling and NLRP3 Inflammasome Activation in the Kidney

**Figure 14:**
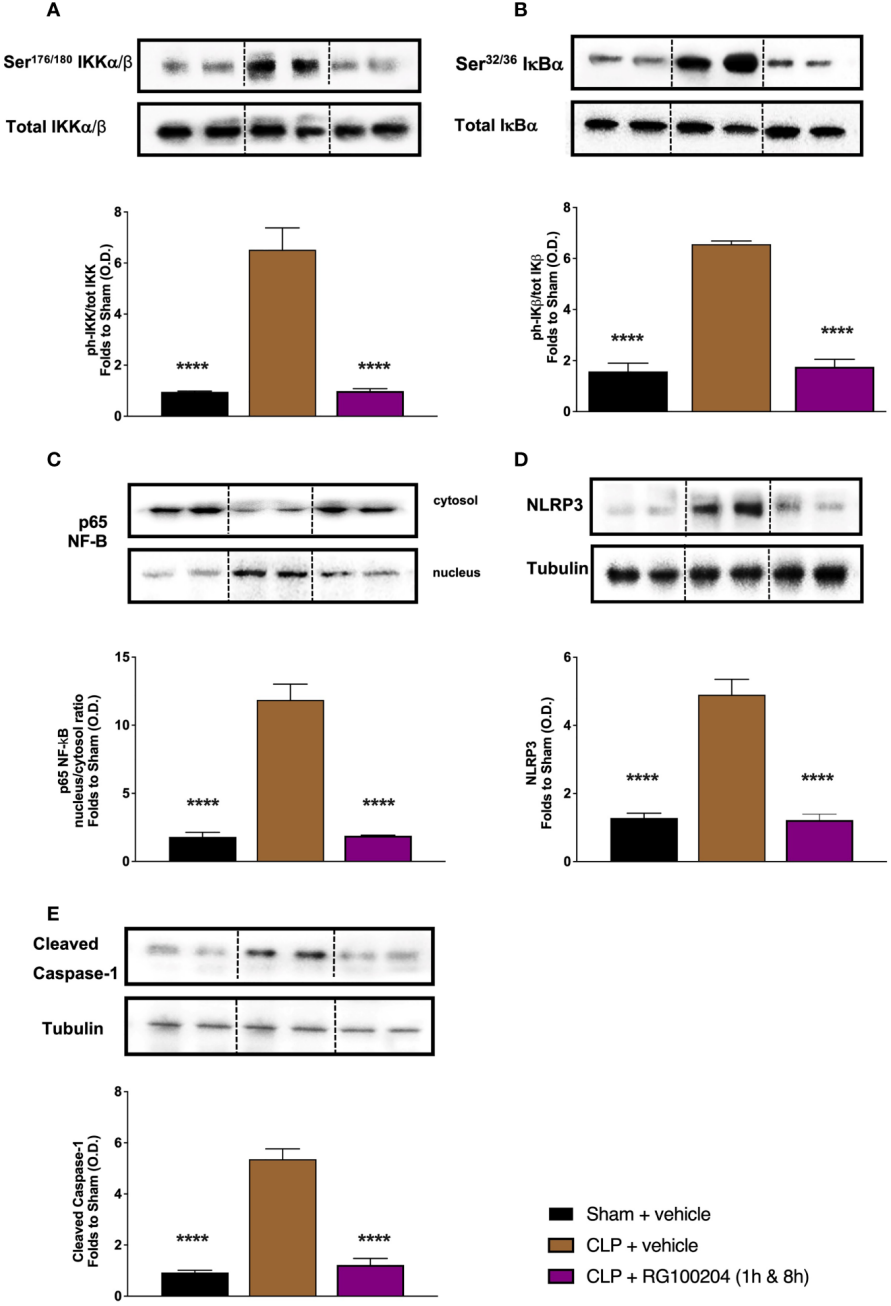
Effect of post-treatment (therapeutic administration) with RG100204 on the NF-ĸB signalling pathway and the activation NLRP3 inflammasome in the kidney. Kidney samples were collected at the end of the experiment and the NF-ĸB signalling pathway, as well as the activation of the NLRP3 inflammasome. Densitometry analysis of the bands is expressed as relative optical density (O.D.) of the **(A)** phosphorylation of IKKα/β at Ser^178/180^ corrected for the corresponding total IKKα/β content and normalized using the related sham band; **(B)** phosphorylation of IĸBα at Ser^32/36^ corrected for the corresponding total IĸBα content and normalized using the related sham band; **(C)** NF-ĸB p65 subunit levels in both, cytosolic and nuclear fractions expressed as a nucleus/cytosol ratio normalized using the related sham bands; **(D)** NLRP3 activation, corrected against tubulin and normalized using the related sham bands; and **(E)** proteolytic cleavage of pro-caspase-1 to activated caspase-1 and normalized using the related sham band. The following groups were studied: sham + vehicle (n = 5), CLP + vehicle (n = 10), CLP + RG100204 (1 h & 8 h) (n = 10). All data were analyzed by one-way ANOVA, followed by a Bonferroni’s *post-hoc* test. Data are expressed as mean ± SEM. ****P < 0.0001 vs. the respective sham-operated group.

When compared to sham-operated mice, mice subjected to CLP and treated with vehicle (control) showed a significant increase in the phosphorylation of IKKα/β at Ser^176/180^, in the phosphorylation of IĸBα at Ser^32/36^ and the translocation of NF-ĸB subunit p65 to the nucleus in the kidney (**Figures 14A–C**). When compared to CLP-animals treated with vehicle, treatment of CLP-animals with RG100204 given 1 h followed by 8 h after CLP surgery significantly attenuated the degree of phosphorylation of IKKα/β at Ser^176/180^, the phosphorylation of IĸBα at Ser^32/36^ and the translocation of the p65 subunit of NF-ĸB to the nucleus in the liver (**Figures 14A–C**), indicating the ability of RG100204 to effectively inhibit the NF-ĸB signalling pathway in the kidney. When compared to sham-operated mice, mice subjected to CLP and treated with vehicle (control) demonstrated a significant increase in the expression of the NLRP3 inflammasome and the cleavage of pro-caspase-1 to caspase-1 in the **liver** (**Figures 14D, E**). However, the activation of the NLPR3 inflammasome was attenuated by the treatment with RG100204 given 1 h & 8 h after CLP surgery (**Figures 14D, E**).

### Effect of Post-Treatment (Therapeutic Administration) With RG100204 on MPO Activity in the Lungs

**Figure 15:**
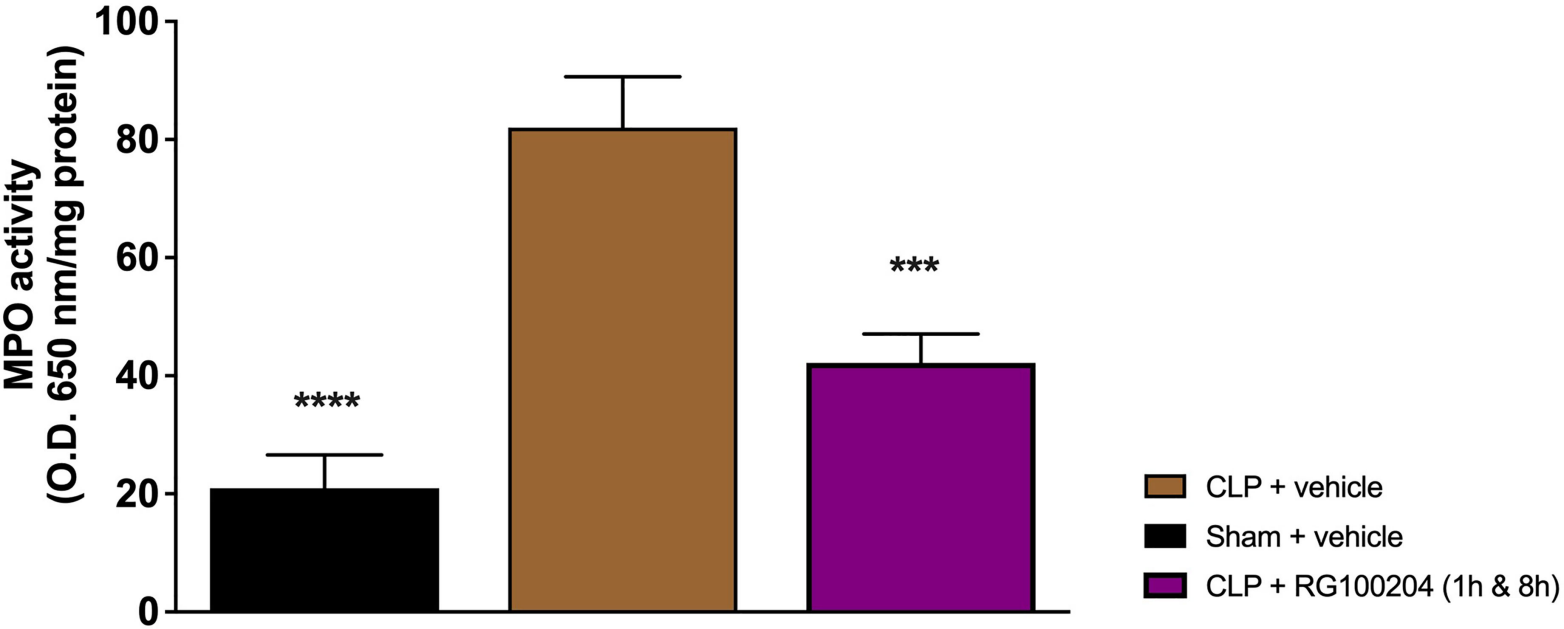
Effect of post-treatment (therapeutic administration) with RG100204 on MPO activity in lung. MPO analysis was conducted on the lungs. The following groups were studied: sham + vehicle (n = 5), CLP + vehicle (n = 10), CLP + RG100204 (1 h & 8 h) (n = 10). All data are expressed as mean ± SEM for n number of observations. All data were analyzed by one-way ANOVA, followed by a Bonferroni’s *post-hoc* test. Data are expressed as mean ± SEM. ***P < 0.001 and ****P < 0.0001 vs. the respective sham-operated group.

When compared to sham-operated mice, mice subjected to CLP and treated with vehicle (control) demonstrated a significant increase in MPO activity in the lungs (**Figure 15**). However, the activity of MPO in the lungs was significantly attenuated by the treatment with RG100204 given 1 h followed by 8 h after CLP surgery (**Figure 15**).

## Discussion

A recent study demonstrated improved survival of *Aqp9^-/-^* knockout mice in an LPS induced mouse model of sepsis (18). While this model is well-suited to study mechanisms of systemic inflammation, which can be harmful when excessive, the LPS model does not include an infecting agent. Inflammation has however evolved to contain infections, and curtailing inflammation in infectious diseases is thus a strategy that may have concomitant beneficial and negative consequences. In the current study we thus asked if loss of *Aqp9* function can confer a net protective effect in a model of sepsis where a bacterial infection is present. This is the first study describing a novel inhibitor, RG100204, that was developed and used for this purpose. In a rat hepatoma cell line RG100204 was able to ameliorate an LPS induced NO and ${\text{O}}_{2}^{-}$ production, thus providing evidence that this substance can reproduce oxide-reducing effects that were previously observed in LPS treated mice (18).

Thus, we utilized RG10024 to further interrogate the potential of AQP9 as a drug target in polymicrobial sepsis. RG100204 administrated as pre- or post-treatment (prophylactic versus therapeutic administration) protected mice from sepsis-induced multiple organ dysfunction (cardiac dysfunction, renal dysfunction, and hepatocellular injury).

A mouse severity scoring system was used as a surrogate marker of mortality in this model of sepsis. Each mouse was assessed at 0 h, 6 h, 18 h, and 24 h after CLP surgery to determine the severity score. The physiological symptoms noted to calculate the score were symptoms of sepsis, which included piloerection, tremors, diarrhoea, respiratory distress, and periorbital exudates. At 24 h, severe sepsis was defined for mice with a severity score of > 3. This was present in CLP-mice treated with vehicle and a score of ≤ 3 in mice administered with RG100204 as a pre and post treatment in a CLP mouse model of sepsis. Body temperature was used as another surrogate marker of mortality. At 24 h, CLP mice treated with vehicle showed a gradual decrease in body temperature that was < 30°C. This suggests that these mice developed severe sepsis. However, at 24 h CLP mice treated with RG100204 as pre-treatment showed a body temperature > 30°C. This suggests that these mice had moderate sepsis and RG100204 treatment was effective at attenuating hypothermia in this CLP-mouse model.

Sepsis results in multiple organ failure and this includes cardiac dysfunction, renal dysfunction, and hepatocellular injury. In this study, pre-treatment of CLP-mice with RG100204 attenuated the cardiac dysfunction (systolic and diastolic), renal dysfunction and hepatocellular injury caused by CLP-sepsis. Hence, the next evaluation was whether the beneficial effect of this drug is maintained when given after the onset of CLP. Post-treatment administration of RG100204 attenuated both the cardiac dysfunction and renal dysfunction but did not significantly reduce the liver injury caused by CLP. However, the most striking finding was that administration as late as 3 h after the onset of polymicrobial sepsis (when followed by further drug administrations at 8 h and in some experiments also 18 h) attenuated the cardiac and renal dysfunction caused by severe sepsis.

What then is the evidence that inhibition of AQP9 exerts beneficial effects in sepsis? At 24 h, cardiac function was assessed by echocardiography investigating both systolic and diastolic function. The effect on systolic function of RG100204 administered pre- and post-CLP surgery was evaluated by measuring % EF, % FS, % FAC, CO and SV. % EF is a common marker used to assess systolic cardiac function, as it reflects cardiac function and remodeling, and it is widely used for diagnostic and prognostic purposes (28). Since the early 1960s, estimated % EF and SV have been measured and both endpoints are now pivotal to the assessment of cardiac function in modern cardiology (28). CLP-sepsis caused a significant decrease in systolic function (as determined by a significant decrease in all parameters of the systolic function measured). In contrast, pre-treatment of CLP-mice with RG100204 attenuated the decline in all parameters used to measure left ventricular systolic function. Similarly, this was demonstrated for all post-treatment dosing times, however none of the dosing times had a significant effect on SV. Moreover, CLP-mice treated with vehicle showed impaired diastolic function which was improved by RG100204 given both as pre- or post-treatment (but not at the latest dosing time), since they exhibited a significant reduction in the mitral valve E/A ratio. The pre- and post-treatment administration (but not at the latest timepoint) of RG100204 attenuated the rise in MPI NFT and MPI IV caused by CLP. The PV VTI and peak velocity are both used to evaluate how efficient blood flows through the pulmonary artery. CLP mice treated with vehicle caused a reduction in PV VTI and peak velocity. Pre-treatment with RG100204 in CLP mice caused a significant increase in PV VTI and peak velocity, therefore the administration of the drug pre-treatment was able to prevent right ventricular dysfunction. However, administration of the drug given post-treatment showed a small but not significant increase in both these parameters. The pre-treatment administration of RG100204 was more effective on systolic and diastolic function compared to post-treatment administration. It must be noted that 1 h administration of drug followed by 8 h administration after CLP was the most effective post-treatment dosing time.

Sepsis is a leading cause for acute kidney injury (29). Serum urea and creatinine are well established biomarkers used to assess renal function. CLP-sepsis was associated with a significant renal dysfunction (rise in both creatinine and urea). Pre-treatment of CLP-animals with RG100204 attenuated the rise in serum creatinine, but not serum urea, caused by sepsis. Most notably, therapeutic administration (post-treatment administration except the latest dosing time) significantly attenuated the renal dysfunction associated with sepsis. Serum levels of urea and creatinine are influenced by other factors (especially in sepsis) and, hence, the evaluation of further biomarkers, such as IL-18 and kidney injury molecule-1 which are markers that have been found to be upregulated early after renal insult in sepsis (30). These are warranted to gain a better insight into the effects of AQP9 inhibition on renal dysfunction in sepsis. In terms of hepatocellular injury, pre- or post-treatment administration of RG100204 in CLP reduced liver injury. Only pre-treatment administration of RG100204 caused a significant reduction in serum ALT but had no effect on AST. None the less ALT is specific for liver injury, while AST is not. However, post-treatment administration had no significant effect on either serum ALT or AST, although in all cases there was a small reduction. Therefore, this provides a good indication that pre-treatment with AQP9 reduced the rise in ALT caused by sepsis and hence, liver injury. LDH is a biomarker used to evaluate tissue damage caused by sepsis. The observed rise in LDH in CLP-sepsis could be due to cellular injury related to bacterial toxins. Increased LDH levels are commonly seen in patients with sepsis, and a rise in LDH has been shown to reflect the degree of tissue damage (31). CLP-sepsis caused a significant increase in serum LDH and, hence, tissue injury. Pre-treatment of CLP-animals with RG100204 and post-treatment (given 1 h followed by 8 h after the onset of sepsis) prevented the rise in LDH caused by sepsis.

Sepsis results in multiple organ failure, including cardiac dysfunction, renal dysfunction, and hepatocellular injury. Taken together, we show that a novel AQP9 inhibitor, RG100204 demonstrates the ability to prevent sepsis-induced multiple organ dysfunction. What, then, is the mechanism by which RG100204 reduces the multiple organ failure associated with sepsis? To gain an insight into the potential mechanism of action of the observed beneficial effects of RG100204 in sepsis, we investigated NF-κB signalling and inflammasome expression in the heart and kidney. NF-κB plays a critical role in sepsis associated multiple organ failure, since NF-κB is involved in regulating the transcription of immunomodulatory mediators involved in the development of sepsis-induced multiple organ failure (32). In this study, we report that CLP mice subjected to polymicrobial sepsis have increased translocation of p65 in both the heart and kidneys which is attenuated by post-treatment with the AQP9 inhibitor RG100204. This is consistent with a recent study demonstrating that *Aqp9* gene deletion reduced NO, ${\text{O}}_{2}^{-}$ , iNOS expression and COX-2 expression through impairment of NF-κB p65 expression/activation in an endotoxemic mouse model. Together, these observations suggest that AQP9 may play a role in the early stage of endotoxic shock, involving the NF-κB pathway. This role could potentially involve AQP9 dependent uptake of extracellular H2O2 (18). Inhibition of NF-κB activation prevents multiple organ injury in animal models of sepsis (33). This shows that NF-κB activation plays a pivotal role in sepsis and provides a potential explanation for the observed reduction in multiple organ injury and dysfunction observed in CLP-mice subjected to RG100204.

Activation of the NLRP3 inflammasome plays a further important role in sepsis (34). Inhibitors of NLRP3 (cortistatin and sulfur dioxide) can attenuate the inflammatory response in mouse models of sepsis and thereby prevent sepsis-induced cardiac dysfunction (35). The NLRP3 inflammasome is required by macrophages to release IL-1β. AQP9 is expressed in leukocytes and activation by an endotoxin challenge results in a modified mRNA expression of *Aqp9* (6). Inhibition of AQPs has been found to prevent inflammasome activation in macrophages (19, 20). Mechanisms involving AQP mediated water or glycerol influx, and cell swelling have been proposed. However, due to relatively large osmotic gradients used in these studies, indirect involvement of AQPs in inflammasome activation should not be discounted at this point. Nevertheless, these findings are consistent with the findings in this study, as we show that activation of the NLRP3 inflammasome (measured as NLRP3 and caspase-1 activation) in the heart and kidney was reduced in CLP mice treated with RG100204. NLRP3 activation involves both priming and triggering signals, hence inhibiting the inflammasome provides beneficial effects for controlling the inflammatory response in sepsis. Thereby, this provides evidence of a role of AQP9 in inflammatory diseases.

Finally, sepsis can affect the lungs, leading to lung failure, which is the most common cause of sepsis induced mortality (36). MPO is an enzyme stored in granules of neutrophils that are released upon neutrophil activation and serves as a marker for inflammation. During an inflammatory response, MPO catalyzes the conversion of chloride and hydrogen peroxide to hypochlorite (37). Neutrophils are involved in sepsis and MPO activity is used to investigate neutrophil infiltration. Uncontrolled migration of neutrophils into the lungs results in MPO sequestration, causing inflammation and lung dysfunction (38). Here we report that CLP mice showed a significant increase in MPO activity in the lungs, which was effectively attenuated by RG100204, hence demonstrating the protective effects of RG100204 on the lungs. This is in agreement with reduced migration of *Aqp9^-/-^* knockout neutrophils in a mouse model of contact hypersensitivity (7).

## Limitations of This Study

Due to restrictions by the held ethical permit, we are unable to measure death as an endpoint. Mortality of animals is not acceptable to used and the “3Rs” in animal research strongly suggest the use of humane endpoints in animal models of sepsis in the UK. Therefore, we use surrogate markers of outcome in the CLP mouse model such as clinical severity score, body temperature, echocardiography, and biochemical markers of organ damage at 24 h after the induction of sepsis to be recorded. We are unable to do longer time evaluations, as this results in significant increase in mortality, which is prohibited by our home office license.

## Conclusion

In conclusion, pre-treatment administration of RG100204 in CLP mice was effective at attenuating hypothermia, systolic and diastolic cardiac dysfunction and reduced renal dysfunction. However, hepatocellular injury was only slightly reduced. Post-treatment of CLP-mice with RG100204 attenuated the cardiac dysfunction (systolic and diastolic), ameliorated renal dysfunction, and reduced the rise in the cell injury marker LDH, which were all caused by CLP-sepsis, but it did not significantly reduce the liver injury and hypothermia. Most importantly, oral administration of RG100204 as late as 3 h after the onset of polymicrobial sepsis (when followed by further drug administrations at 8 h and in some experiments also 18 h) attenuated the cardiac and renal dysfunction caused by severe sepsis. RG100204 also demonstrated the ability to attenuate the activation of NF-ĸB as well as the expression of the NLRP3 inflammasome in the heart and kidney. Taken together our findings suggests that AQP9 inhibitors may hold promise for the use in sepsis.

## Data Availability Statement

The original contributions presented in the study are included in the article/**Supplementary Material**. Further inquiries can be directed to the corresponding authors.

## Ethics Statement

All animal protocols in this study were reviewed and approved by the Animal Use and Care Committee of Queen Mary University of London (QMUL), in accordance with the Home Office Guidance on the Operation of Animals (Scientific Procedure Act 1986), published by Her Majesty’s Stationary Office and the Guide for the Care and Use of Laboratory Animals of the National Research Council and were reviewed and approved by the Animal Welfare Ethics Review Board of QMUL.

## Author Contributions

SM, CO’R, EA, GA, MR, KD, AT, and PG performed the experiments. SM, CT, MR, AT, JE, and GC planned experiments. SM, CO’R, MC, EA, and GA analyzed the data. SM, CT, and MR contributed to the writing of the manuscript. All authors contributed to the article and approved the submitted version.

## Funding

SM is supported by a MRes/PhD-Studentship awarded by the British Heart Foundation (BHF) (Award number: FS/17/69/33484). This work was, in part, supported by a commercial grant awarded to William Harvey Research Limited by Apoglyx, and (in part) by a grant from the William Harvey Research Foundation. GC was supported by a grant from the Italian Government (“Fondo Integrativo Speciale per la Ricerca 2020” - FISR 2020 CoVAPin, grant # FISR2020IP_04051).

## Conflict of Interest

Author MR is an employee of ApoGlyx AB, which is pursuing AQP9 inhibitors for commercial applications. Authors KD and JE declare that they are employees of Red Glead Discovery AB, which is a shareholder of ApoGlyx AB. This study received funding from Apoglyx. The funder had the following involvement with the study: Study design, data collection and analysis (*in vitro* data only, see **Figure 1** and **2**) and the presentation of the manuscript.

The remaining authors declare that the research was conducted in the absence of any commercial or financial relationships that could be construed as a potential conflict of interest.

## Correction Note

A correction has been made to this article. Details can be found at: fimmu.2025.1672460.

## Publisher’s Note

All claims expressed in this article are solely those of the authors and do not necessarily represent those of their affiliated organizations, or those of the publisher, the editors and the reviewers. Any product that may be evaluated in this article, or claim that may be made by its manufacturer, is not guaranteed or endorsed by the publisher.

## Supplementary Material

Supplementary Figure 1 Effect of RG100204 on LPS-induced viability of FaO cells. Cultured FaO cells were treated with the vehicle alone (1% DMSO; Ctrl) or with a series of doses of RG100204 in presence or absence of LPS (1 µg/mL) for 6 h. Cell viability was measured by MTT assay. The effect of each concentration of RG100204 is compared to the corresponding basal condition (1% DMSO) in presence or absence of LPS. Data were analyzed by one-way ANOVA by Tukey’s post-hoc test. Values are expressed as mean ± SD; n = 6. **P < 0.01.
