# Correction: RG100204, a novel Aquaporin-9 inhibitor, reduces septic cardiomyopathy and multiple organ failure in murine sepsis

**DOI:** 10.3389/fimmu.2025.1672460

**Published:** 2025-08-21

**Authors:** Shireen Mohammad, Caroline E. O’Riordan, Chiara Verra, Eleonora Aimaretti, Gustavo Ferreira Alves, Klaus Dreisch, Johan Evenäs, Patrizia Gena, Angela Tesse, Michael Rützler, Massimo Collino, Giuseppe Calamita, Christoph Thiemermann

**Affiliations:** ^1^ William Harvey Research Institute, Queen Mary University of London, London, United Kingdom; ^2^ Department of Clinical and Biological Sciences, University of Turin, Turin, Italy; ^3^ Department of Neurosciences “Rita Levi Montalcini”, University of Turin, Turin, Italy; ^4^ Red Glead Discovery Akiebolag (AB), Lund, Sweden; ^5^ Department of Biosciences, Biotechnologies and Biopharmaceutics, University of Bari “Aldo Moro”, Bari, Italy; ^6^ Nantes Université, Instite National de la Santé et de la Recherche Médicale (INSERM), Centre National de la Rescherche Scientifique (CNRS), l’institut du Thorax, Nantes, France; ^7^ Department of Biochemistry and Structural Biology, Lund University, Lund, Sweden; ^8^ Apoglyx Akiebolag (AB), Lund, Sweden

**Keywords:** aquaporin (AQP), sepsis, cecal ligation and puncture, inflammation, multiple organ failure

There was an error in **Figure 13** as published. The representative images related to Panel A have been inadvertently duplicated in Panel E. The corrected **Figure 13** and its caption appear below.

**Figure 13:**
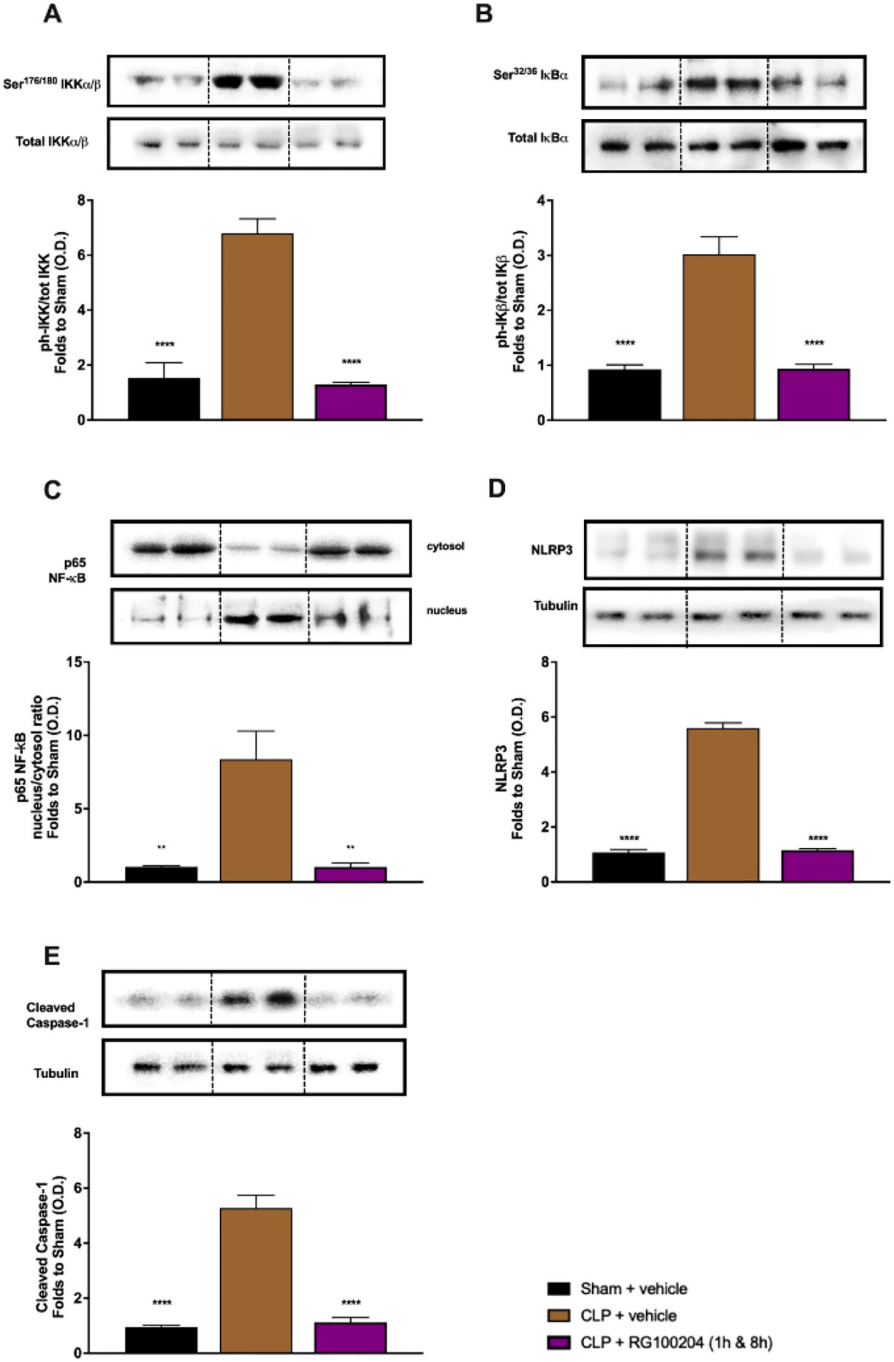
Effect of post-treatment (therapeutic administration) with RG100204 on the NF-ĸB signalling pathway and the activation NLRP3 inflammasome in the heart. Heart samples were collected at the end of the experiment and the NF-kB signalling pathway, as well as the activation of the NLRP3 inflammasome. Densitometry analysis of the bands is expressed as relative optical density (O.D.) of the **(A)** phosphorylation of IKKa/b at Ser178/180 corrected for the corresponding total IKKa/b content and normalized using the related sham band; **(B)** phosphorylation of IĸBa at Ser32/36 corrected for the corresponding total IĸBa content and normalized using the related sham band; **(C)** NF-ĸB p65 subunit levels in both, cytosolic and nuclear fractions expressed as a nucleus/cytosol ratio normalized using the related sham bands; **(D)** NLRP3 activation, corrected against tubulin and normalized using the related sham bands; and **(E)** proteolytic cleavage of pro-caspase-1 to activated caspase-1 and normalized using the related sham band. The following groups were studied: sham + vehicle (n = 5), CLP + vehicle (n = 10), CLP +RG100204 (1 h & 8 h) (n = 10). All data were analyzed by one-way ANOVA, followed by a Bonferroni’s post-hoc test. Data are expressed as mean ± SEM. **P < 0.01 and ****P < 0.0001 vs. the respective sham-operated group.

The original article has been updated.

