# Supplementary figures and images for: RG100204, A Novel Aquaporin-9 Inhibitor, Reduces Septic Cardiomyopathy and Multiple Organ Failure in Murine Sepsis
Source: Front Immunol. 2022 Jun 14;13:900906. doi: 10.3389/fimmu.2022.900906 (PMC9238327; doi:10.3389/fimmu.2022.900906)

**
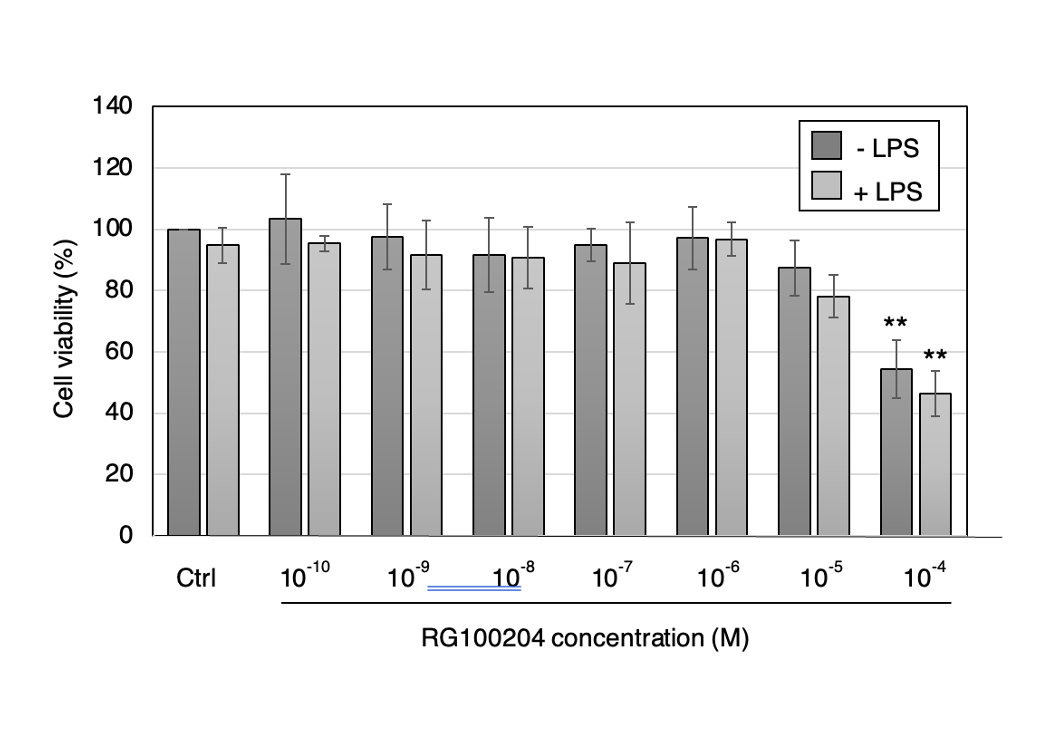
Supplementary Figure 1:**

Supplement: Supplementary Figure 1 — Effect of RG100204 on LPS-induced viability of FaO cells. Cultured FaO cells were treated with the vehicle alone (1% DMSO; Ctrl) or with a series of doses of RG100204 in presence or absence of LPS (1 µg/mL) for 6 h. Cell viability was measured by MTT assay. The effect of each concentration of RG100204 is compared to the corresponding basal condition (1% DMSO) in presence or absence of LPS. Data were analyzed by one-way ANOVA by Tukey’s post-hoc test. Values are expressed as mean ± SD; n = 6. **P < 0.01. [file DataSheet_1.docx]
